# The Na^+^,K^+^,2Cl^−^ Cotransporter, Not Aquaporin 1, Sustains Cerebrospinal Fluid Secretion While Controlling Brain K^+^ Homeostasis

**DOI:** 10.1002/advs.202409120

**Published:** 2024-12-18

**Authors:** Dennis Bo Jensen, Trine L. Toft‐Bertelsen, Dagne Barbuskaite, Jane Stubbe, Sandor Nietzsche, Tenna Capion, Nicolas H. Norager, Markus H. Olsen, Andreas T. Sørensen, Henrik Dimke, Christian A. Hübner, Marianne Juhler, Nanna MacAulay

**Affiliations:** ^1^ Department of Neuroscience University of Copenhagen Blegdamsvej 3 Copenhagen N 2200 Denmark; ^2^ Department of Molecular Medicine University of Southern Denmark Campusvej 55 Odense 5230 Denmark; ^3^ Center for Electron Microscopy Jena University Hospital Ziegelmühlenweg 1 07743 Jena Germany; ^4^ Department of Neurosurgery University Hospital of Copenhagen – Rigshospitalet Blegdamsvej 9 Copenhagen 2100 Denmark; ^5^ Department of Neuroanaesthesiology University Hospital of Copenhagen – Rigshospitalet Blegdamsvej 9 Copenhagen 2100 Denmark; ^6^ Department of Nephrology Odense University Hospital J.B. Winsløws Vej 4 Odense 5000 Denmark; ^7^ Hübner Institute of Human Genetics Jena University Hospital Am Klinikum 1 07747 Jena Germany; ^8^ Department of Clinical Medicine University of Copenhagen Blegdamsvej 3 Copenhagen 2200 Denmark

**Keywords:** aquaporin, choroid plexus, CSF, hydrocephalus, K^+^, NKCC1

## Abstract

Disturbances in the brain fluid balance can lead to life‐threatening elevation in intracranial pressure (ICP), which represents a vast clinical challenge. Targeted and efficient pharmaceutical therapy of elevated ICP is not currently available, as the molecular mechanisms governing cerebrospinal fluid (CSF) secretion are largely unresolved. To resolve the quantitative contribution of key choroid plexus transport proteins, this study employs mice with genetic knockout and/or viral choroid plexus‐specific knockdown of aquaporin 1 (AQP1) and the Na^+^, K^+^, 2Cl^−^ cotransporter 1 (NKCC1) for in vivo determinations of CSF dynamics, ex vivo choroid plexus for transporter‐mediated clearance of a CSF K^+^ load, and patient CSF for [K^+^] quantification. CSF secretion and ICP management occur independently of choroid plexus AQP1 expression, whereas both parameters are reduced by 40% upon choroid plexus NKCC1 knockdown. Elevation of [K^+^]_CSF_ increases the choroid plexus Na^+^/K^+^‐ATPase activity, and favors inwardly‐directed net NKCC1 transport, which, together, promote CSF K^+^ clearance, while maintaining undisturbed CSF secretion rates. CSF from patients with post‐hemorrhagic hydrocephalus does not display elevated [K^+^]_CSF_, suggesting that NKCC1 maintains net outward transport direction during post‐hemorrhagic hydrocephalus formation. Direct or indirect therapeutic modulation of choroid plexus NKCC1 can thus be a potential promising pharmacological approach against brain pathologies associated with elevated ICP.

## Graphical abstract

CSF is secreted by the choroid plexus in each ventricle. The choroid plexus water channel, aquaporin 1, is not required for CSF secretion and thus brain fluid dynamics, whereas the Na^+^, K^+^, 2Cl^‐^ cotransporter 1 is a key factor in this process. Elevated ventricular K^+^ alters the transport activity to clear brain K^+^ while maintaining the CSF secretion rate.

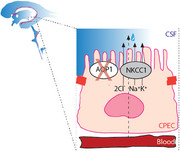

## 1 Introduction

The mammalian brain is immersed in the cerebrospinal fluid (CSF) that protects the brain from mechanical insult and serves as a transport route for nutrients and metabolites between cells and structures in the brain. The CSF is continuously produced at a rate of ≈500 mL day^−1^ in adult humans,^[^ ^1^ ^]^ predominantly by the choroid plexus.^[^ ^2^, ^3^, ^4^, ^5^ ^]^ The choroid plexus is a specialized secretory epithelium in the brain ventricles, supporting secretion of electrolytes and associated fluid from the blood to the ventricular compartment by its high metabolic rate, its microvilli‐covered luminal surface, and its polarized expression of transport proteins.^[^ ^4^ ^]^ Pharmacological inhibition of transport proteins underlying CSF secretion modulates the intracranial pressure (ICP) in rodents and humans.^[^ ^1^, ^6^, ^7^, ^8^, ^9^ ^]^ Thus, such an approach could be employed as a complementary therapeutic treatment option in some of the many conditions encompassing pathological brain fluid accumulation and elevated ICP, i.e., normal pressure hydrocephalus (NPH), posthemorrhagic hydrocephalus (PHH), and idiopathic intracranial hypertension (IIH). These conditions are prevalent in distinct age groups, with an estimated 1–2% of elderly patients suffering from NPH,^[^ ^10^, ^11^ ^]^ up to 0.1% of the pediatric population experiencing PHH,^[^ ^12^, ^13^ ^]^ and 0.02% of young women diagnosed with IIH.^[^ ^14^ ^]^ If left untreated, the elevated ICP may cause neurological compromise, coma, and ultimately may be fatal to the patient.^[^ ^15^ ^]^ First‐line treatments of pressure‐related brain disorders often relies on neurosurgical diversion of excess CSF, such as surgical implantation of a ventriculo‐peritoneal shunt (diversion of excess ventricular fluid to the peritoneal cavity),^[^ ^16^ ^]^ endoscopic third ventriculostomy (puncture of the third ventricle floor),^[^ ^17^ ^]^ or even choroid plexus cauterization,^[^ ^18^ ^]^ which, although lifesaving, may associate with complications, high failure rates, and patient morbidity.^[^ ^19^, ^20^ ^]^

Identification of the molecular mechanisms supporting CSF secretion is key for future rational design of pharmacological approaches to manage ICP, a priority for hydrocephalus patients and relatives.^[^ ^21^ ^]^ Aquaporin 1 (AQP1) is highly expressed in the luminal membrane of the choroid plexus,^[^ ^22^ ^]^ and has been proposed to be involved in CSF secretion.^[^ ^23^ ^]^ However, it remains unresolved whether the reported small reduction in CSF secretion in AQP1 knockout mice occurred indirectly via the disturbed systemic fluid homeostasis that may arise with deletion of AQP1 in the kidney,^[^ ^24^ ^]^ since (*i*) humans deficient in AQP1 report no neurological deficit^[^ ^25^, ^26^ ^]^ and (*ii*) CSF secretion readily takes place against an osmotic gradient,^[^ ^9^, ^27^, ^28^, ^29^ ^]^ which is irreconcilable with AQP‐dependent osmotic water flux across the choroid plexus (for review, see^[^ ^4^ ^]^).

The Na^+^‐HCO_3_^−^ import protein (NCBE) in the basolateral membrane, and the Na^+^/K^+^‐ATPase, the Na^+^‐dependent bicarbonate cotransporter (NBCe2), and the Na^+^, K^+^, 2Cl^−^ cotransporter (NKCC1) in the luminal membrane together contribute to CSF secretion.^[^ ^9^, ^30^, ^31^, ^32^, ^33^, ^34^ ^]^ Pharmacological inhibition of NKCC1 in healthy rodents decreases the CSF secretion rate^[^ ^9^, ^32^, ^33^, ^35^ ^]^ and lowers the ICP.^[^ ^9^ ^]^ However, it remains unresolved whether NKCC1 contributes to net CSF secretion by transporting ions and fluid from the choroid plexus epithelium to the ventricles^[^ ^9^, ^35^, ^36^, ^37^ ^]^ or by transporting ions and fluid from the ventricles to the cell interior.^[^ ^38^, ^39^ ^]^ Inwardly‐directed net NKCC1 transport may be promoted by a proposed elevation of CSF K^+^ concentration ([K^+^]_CSF_) in connection with brain hemorrhage and ensuing PHH formation,^[^ ^40^, ^41^ ^]^ although a brain hemorrhage‐induced elevation of [K^+^]_CSF_ in patients remains to be demonstrated, as does a [K^+^]_CSF_ –dependent modulation of the CSF secretion rate.

We here resolve the contribution of AQP1 and NKCC1 to CSF secretion in physiology with genetic knockout and viral knockdown approaches in combination with in vivo determination of CSF dynamics and choroid plexus function in steady state and during mimicked conditions of CSF [K^+^] excess, the latter of which was not detected in patients with PHH.

## 2 Results

### 2.1 Undisturbed Whole‐Body Water Homeostasis in AQPqp1^−/−^ Mice Despite Elevated Water Intake

**Figure 1:**
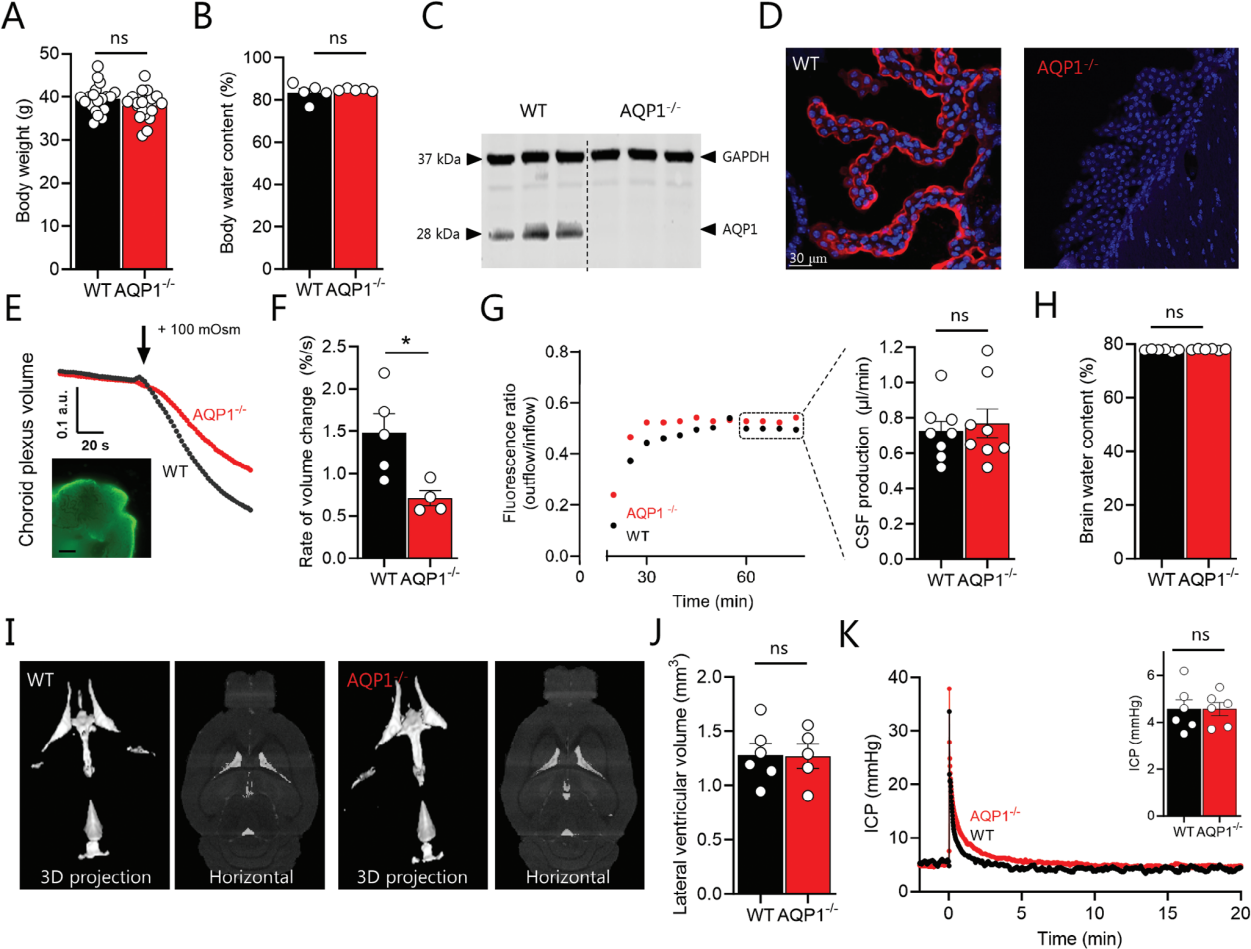
Knockout of AQP1 reduces choroid plexus osmotic water permeability but not CSF secretion or ICP. A) Body weight (n = 20) and B) body water content (n = 5) of wildtype (WT) and AQP1 knockout (AQP1^−/−^) mice. C) Representative Western blot of lysates from mouse choroid plexus from WT and AQP1^−/−^ mice (AQP1: 28 kDa; GAPDH: 37 kDa). D) Representative immunolabeling of AQP1 (red; DAPI in blue) of choroid plexus from WT and AQP1^−/−^ mice. E) Representative volume response to an osmotic challenge of +100 mOsm (indicated by arrow) in WT and AQP1^−/−^ choroid plexus loaded with the fluorescent dye calcein (example micrograph shown in inset, scale bar = 60 µm) with quantification of the rate of volume changes in (F), n = 5 WT and n = 4 AQP1^−/−^. G) Representative ventriculo‐cisternal perfusion time course of the dextran ratio (outflow/inflow) in a WT and AQP1^−/−^ mouse with summarized CSF production rates, obtained from the average of the time points indicated in a dashed box, n  =  8 of each. H) Brain water content in WT and AQP1^−/−^ mice, n  =  6. I) Representative MRI scans of WT and AQP1^−/‐^mice (3D projections and horizontal plane) with J) lateral ventricular volume quantification, n = 6 WT and 5 AQP1^−/−^. K) Representative intracranial pressure (ICP) trace obtained in WT and AQP1^−/‐^ mice, with summarized ICP values illustrated in inset, n  =  6 of each. Statistical evaluation with Student's t‐test. ^*^; *P* <0.05, ns; not significant.

To determine the role of AQP1 in CSF dynamics, we initially employed the constitutive AQP1^−/−^ mouse model with systemic deficiency in the AQP1 protein. The AQP1 knockout was genotypically verified but conferred no apparent behavioral phenotype to the mice, nor a difference in bodyweight (38.0 ± 0.7 g for AQP1^−/−^ vs 39.5 ± 0.7 g for WT, n = 20, *P* = 0.16, **Figure** **1**A). Despite the widespread expression of AQP1 in the body, including lung epithelium and kidney proximal tubules, thin ascending limb of Henle, and vasa recta,^[^ ^42^ ^]^ whole‐body MRI determination of systemic water homeostasis revealed similar body water percentage in AQP1^−/−^ mice (84.8 ± 0.3%) and WT mice (83.5 ± 1.9%, n = 5, *P* = 0.54, Figure 1B). The undisturbed whole body water balance was sustained despite the AQP1^−/−^‐dependent increase in diuresis, due to the associated elevated water intake by AQP1^−/−^ mice versus WT mice (Figure S1A–D, Supporting Information and ^[^ ^24^, ^43^, ^44^ ^]^).

### 2.2 AQP1 Contributes Osmotic Water Permeability to the Choroid Plexus but not to CSF Secretion

The high expression of AQP1 in the luminal membrane of WT choroid plexus was completely absent in the AQP1^−/−^ mice (Figure 1C,D; see Figure S1E, Supporting Information for entire Western blot). To assess the osmotic water permeability of the AQP1‐expressing and ventricle‐facing membrane, ex vivo mouse choroid plexus loaded with the fluorescent dye calcein was monitored by live imaging during exposure to an abrupt hyperosmotic challenge. A hyperosmotic gradient of 100 mOsm was introduced by a swift replacement of the artificial cerebrospinal fluid (aCSF) with one containing additional 100 mm mannitol. This resulted in choroid plexus shrinkage in tissue from both WT and AQP1^−/−^ mice (Figure 1E), although at a slower rate in choroid plexus obtained from the AQP1^−/−^ mice (0.71 ± 0.08%/s, n = 4) as compared to those obtained from the WT mice (1.73 ± 0.31%/s, n = 5, *P* < 0.05, Figure 1F). These data indicate that AQP1 contributes ≈60% of the osmotic water permeability of the luminal membrane of the choroid plexus epithelium. To determine whether the AQP1‐mediated osmotic water permeability contributes to net CSF secretion, we employed the ventriculo‐cisternal perfusion assay to assess the CSF secretion rate in anesthetized and mechanically ventilated mice. This assay relies on slow infusion of equiosmolar, gas‐equilibrated, 37° C aCSF containing a fluorescent dye into the lateral ventricle, with simultaneous fluid collection from a cisterna magna puncture. The dilution of the fluorescent dye, monitored as a function of time, originates from endogenous fluid secretion into the ventricular system, and is employed to calculate the rate of CSF secretion.^[^ ^35^ ^]^ The CSF secretion rate was similar in the two genotypes (0.77 ± 0.08 µL min^−1^ in AQP1^−/−^ mice vs 0.72 ± 0.06 µL min^−1^ in WT, n = 8, *P* = 0.66, Figure 1G), indicating that AQP1 is not required for net CSF secretion in mice.

### 2.3 AQP1 is not Required for Total Brain Water Distribution or ICP Maintenance

To determine if the overall CSF dynamics were altered with the absence of AQP1, we assessed the total brain fluid content and its intracerebral distribution. The total brain water was quantified with the wet‐dry technique, which illustrated similar percentage water content in the two genotypes (78.0 ± 0.1% in the AQP1^−/−^ mice vs 77.8 ± 0.2% in the WT mice, n = 6, *P* = 0.24, Figure 1H). MRI analysis demonstrated a similar distribution of the brain fluids (Figure 1I) with no significant difference in lateral ventricular volume (1.27 ± 0.11 mm^3^ in the AQP1^−/−^ mice, n = 5 vs 1.28 ± 0.11 mm^3^ in the WT mice, n = 6, *P* = 0.95, Figure 1J) or in any other CSF compartments (Figure S1F–H, Supporting Information). The constant brain fluid content was reflected in an undisturbed ICP in the AQP1^−/−^ mice (4.6 ± 0.3 mmHg vs 4.6 ± 0.4 mmHg in the WT, n = 6, *P* = 0.99, Figure 1K). Altogether, these data suggest that AQP1 is not required for CSF secretion and thus total brain water dynamics.

### 2.4 Undisturbed Brain Water Homeostasis After Viral Knockdown of AQP1 in the Choroid Plexus

**Figure 2:**
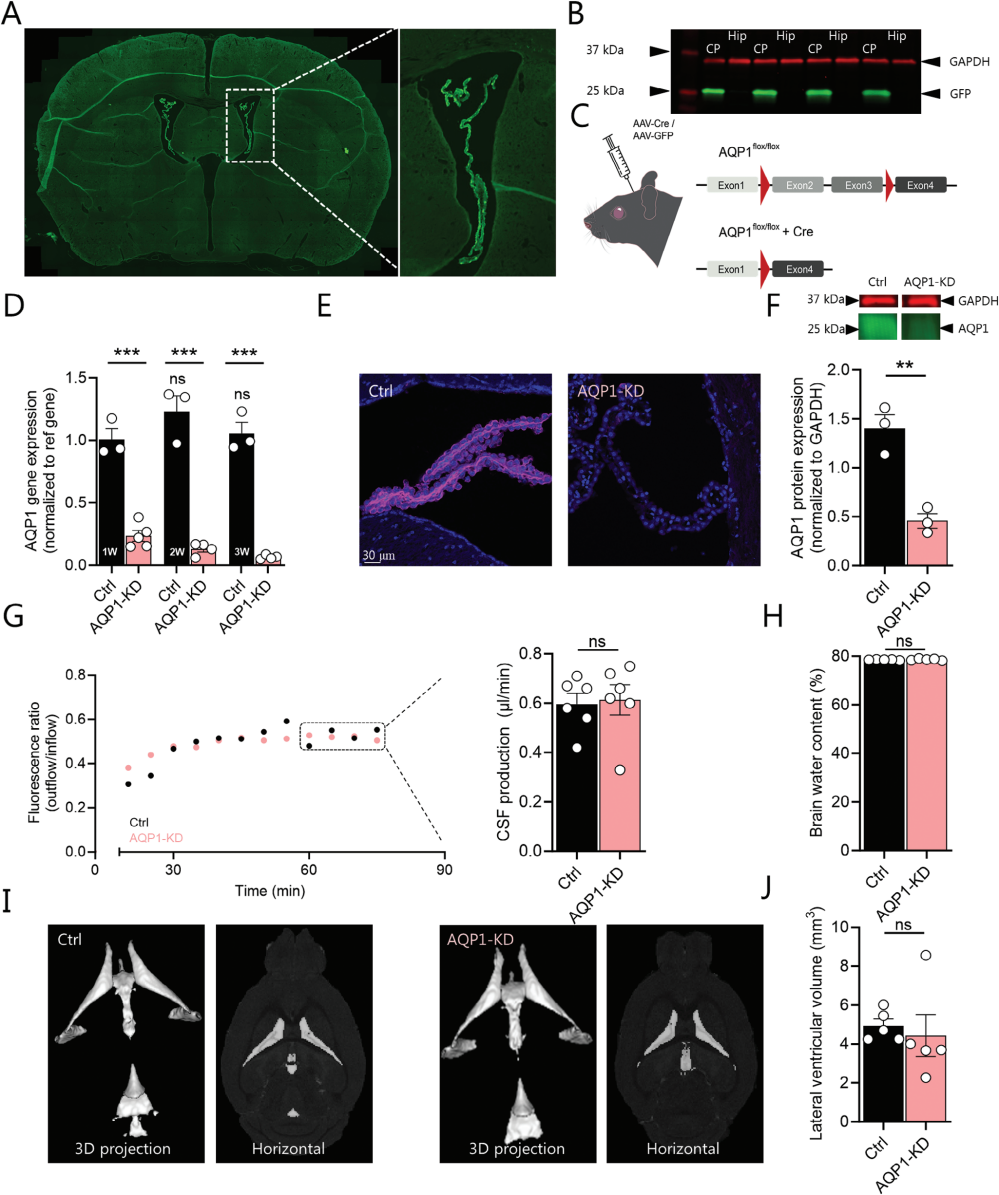
Viral knockdown of choroid plexus AQP1 does not modulate CSF dynamics. A) Representative targeting of GFP (green) to the mouse choroid plexus after intraventricular delivery of AAV5‐GFP. B) Western blotting of choroid plexus (CP) and hippocampal (Hip) lysates after intraventricular delivery of AAV5‐GFP (GFP, green, 25 kDa; GAPDH, red, 37 kDa), n = 4 mice. C) Schematic of the AAV‐Cre approach employed to knock down AQP1 in choroid plexus of AQP1^flox/flox^ mice. D) mRNA expression levels of AQP1 normalized to a reference gene in mouse choroid plexus from control (AAV‐GFP‐injected) and AQP1‐KD (AAV‐Cre‐injected) mice 1–3 weeks (1‐3 W) post‐injection, n = 3 control, n = 5 AQP1‐KD, 1 week, n = 4 AQP1‐KD, 2–3 weeks, ns above the bars refers to lack of statistical difference of controls compared to week 1 and lines above the histograms refer to statistical difference between control and AQP1‐KD mice. E) Representative immunolabeling of AQP1 (magenta, DAPI in blue) in choroid plexus of a control (AAV‐GFP) and an AQP1‐KD (AAV‐Cre) mouse, n = 3 of each. F) Choroid plexus AQP1 protein expression of AQP1‐KD and control mice 3 weeks post‐injection with Western blot of AQP1 (green, 28 kDa) and GAPDH (red, 37 kDa) in insert above the histogram, representative experiment with n = 3 mice (out of 3 independent experiments, see Figure S2G, Supporting Information, for other Western blots). G) Representative ventriculo‐cisternal perfusion time course of the dextran ratio (outflow/inflow) in a control (AAV‐GFP) and an AQP1‐KD (AAV‐Cre) mouse three weeks after injection, with summarized CSF production rates, obtained from the average of the time points indicated in a dashed box, n  =  6 of each. H) Brain water content of control (AAV‐GFP) and AQP1‐KD (AAV‐Cre) mice, 3 weeks post‐injection, n  =  5 of each. I) Representative MRI brain scans of control (AAV‐GFP) and AQP1‐KD (AAV‐Cre) mice, 3 weeks post‐injection (3D projections and horizontal) with J) lateral ventricular volume quantification, n = 5 of each. Statistical evaluation with one‐way ANOVA with Sidak's multiple comparison post hoc test (panel D) or Student's t‐test (remaining panels). ^**^; *P* < 0.01, ^***^; *P* < 0.001, ns; not significant.

To target AQP1 exclusively in the choroid plexus in the adult mouse, we employed intraventricular delivery of an AAV serotype 5 vector, which efficiently targets epithelia.^[^ ^45^, ^46^, ^47^ ^]^ To initially demonstrate preferential viral transduction of the choroid plexus, we employed an AAV encoding green fluorescent protein (GFP) stereotactically delivered into the lateral ventricle of anaesthetized mice. Subsequent western blotting and immunohistochemical analysis of the mouse brains two weeks post‐injection demonstrated preferential targeting of the vector to the choroid plexus epithelium (**Figure** **2**A) with complete absence of GFP expression in the hippocampus neighboring the ventricles (Figure 2B; see Figure S2A, Supporting Information, for entire Western blot). To knock down AQP1 selectively in choroid plexus, we injected the AAV containing GFP fused to Cre recombinase into the lateral ventricle of mice possessing loxP sites flanking exon 2 and 3 of the AQP1 gene (AQP1^flox/flox^), henceforward termed “AQP1‐KD” (Figure 2C). AQP1^flox/flox^ mice injected with GFP‐containing AAV lacking Cre recombinase served as control, henceforward termed “control”. To determine the temporal efficiency of this approach, we performed qPCR on choroid plexus excised from the mice one, two, and three weeks after AAV injection. The AQP1‐coding transcript was significantly reduced already one week after virus injection (78% after one week, 89% after two weeks, 92% after three weeks, n = 3 control, n = 5 AQP1‐KD 1 week, n = 4 AQP1‐KD 2–3 weeks, *P* < 0.001, Figure 2D). Parallel qPCR targeted toward transcripts encoding other transporters involved in CSF secretion (*ATP1A1*, *SLC12A2*, *SLC4A2*, *SLC4A5*, *SLC4A10*) demonstrated that their abundance remained undisturbed (Figure S2B–F, Supporting Information). The choroid plexus AQP1 protein expression was robustly reduced following the AAV injection, as visualized with near absence of AQP1 in immunohistochemical staining (n = 3 mice, see representative micrograph in Figure 2E) and quantified with Western blotting, which demonstrated a 62 ± 6% reduction of AQP1 in choroid plexus from AQP1‐KD (AAV‐Cre‐injected) mice compared to choroid plexus from control (AAV‐GFP‐injected) mice three weeks post‐injection (n = 3 independent experiments, each with 2–5 biological replicates, see representative experiment in Figure 2F, and all Western blots in Figure S2G, Supporting Information). Western blot analysis of kidney tissue obtained at the same post‐injection time point demonstrated that AQP1 expression remained undisturbed in this organ following the intracerebroventricular delivery of the Cre‐containing vector (Figure S2K, Supporting Information).

### 2.5 Viral Knockdown of AQP1 in the Choroid Plexus Does Not Modulate CSF Dynamics

The CSF secretion rate was similar in AQP1‐KD (0.61 ± 0.06 µL min^−1^, n = 6) and control (0.60 ± 0.04 µL min^−1^, n = 6, *P* = 0.83, Figure 2G) mice, as was their total brain water content (78.6 ± 0.2% in AQP1‐KD mice versus 78.5 ± 0.1% in control mice, n = 5, *P* = 0.61, Figure 2H). The ventricular size was undisturbed following knockdown of AQP1 (Figure 2I) with lateral ventricle volumes of 4.44 ± 1.07 mm^3^, n = 5 in the AQP1‐KD mice versus 4.94 ± 0.35 mm^3^ in the control mice, n = 5, *P* = 0.67 (Figure 2J, see Figure S2H–J (Supporting Information) for the volume of the remaining CSF compartments). These findings support the notion of AQP1 not being required for CSF secretion in mice under physiological conditions or for the basic CSF homeostasis in the murine brain.

### 2.6 Viral Knockdown of NKCC1 in the Choroid Plexus Reduces CSF Secretion, ICP, and Ventricular Volume

**Figure 3:**
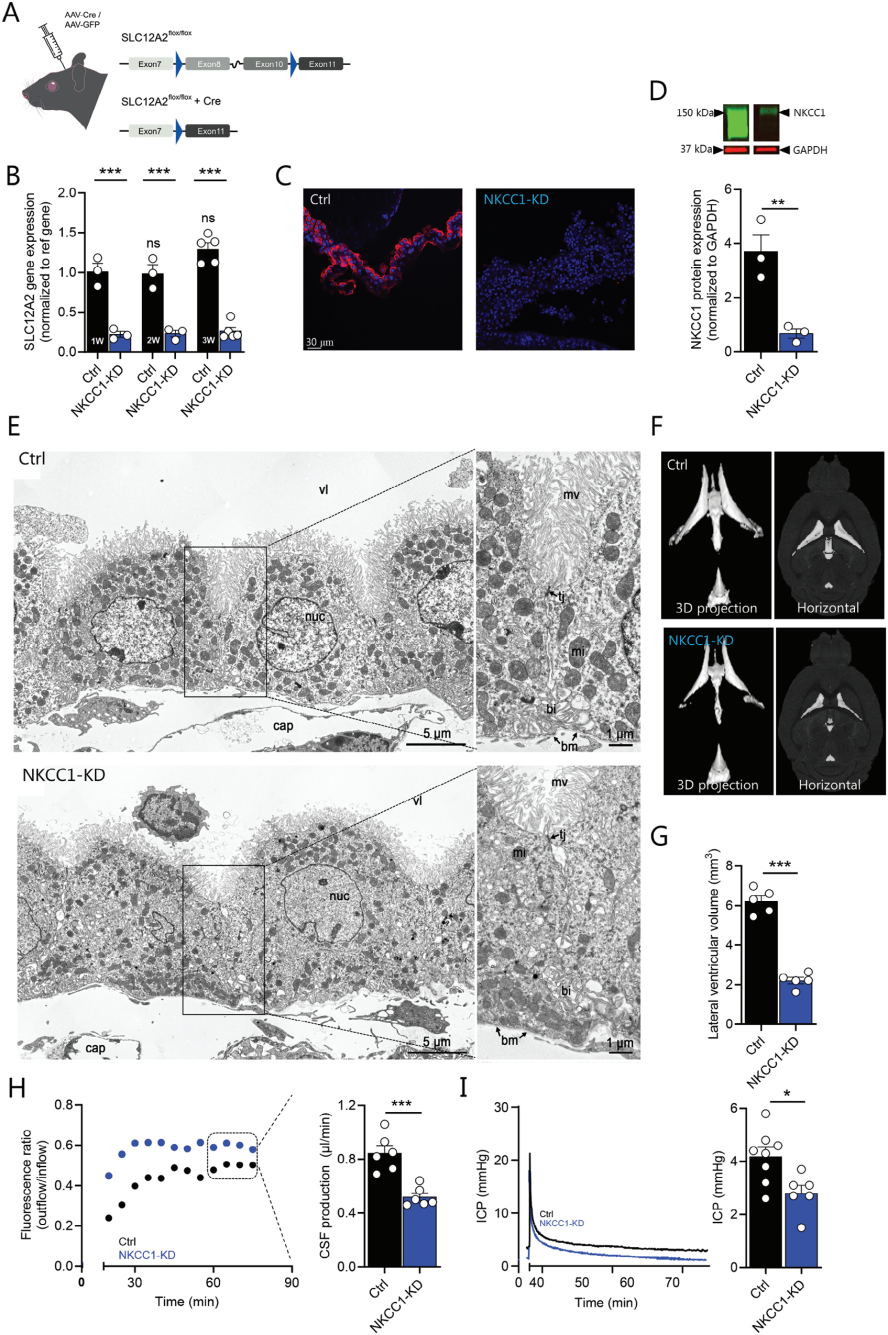
Viral knockdown of NKCC1 in the choroid plexus reduces CSF secretion and ventricular volume. A) Schematic of the AAV‐Cre approach employed to knock down NKCC1 in choroid plexus of SLC12A2^flox/flox^ mice. B) mRNA expression levels of NKCC1 normalized to reference genes in mouse choroid plexus from control (AAV‐GFP‐injected) and NKCC1‐KD (AAV‐Cre‐injected) mice 1–3 weeks (1‐3 W) after injection, n = 3 for 1–2 weeks and n = 5 for 3 weeks, ns above the bars refers to lack of statistical difference of controls compared to week one and lines above the histograms refer to statistical difference between control and NKCC1‐KD mice. C) Representative immunolabeling of NKCC1 (red, DAPI in blue) in choroid plexus of control and NKCC1‐KD mice, n = 3 of each. D) Choroid plexus NKCC1 protein expression of NKCC1‐KD and control mice three weeks after injection with Western blot of NKCC1 (green, 150 kDa) and GAPDH (red, 37 kDa) in insert above the histogram, representative experiment with n = 3 mice (out of 3 independent experiments, see Figure S3E (Supporting Information) for other Western blots). E) Representative electron microscopy micrographs of choroid plexus from control and NKCC1‐KD mice. mv: microvilli; tj: tight junction; bm: basal membrane; bi: basolateral infoldings; mi: mitochondria; nuc: nucleus; vl: ventricular lumen; cap: blood capillary. F) Representative MRI brain scans of control and NKCC1‐KD mice, three weeks post‐AAV injection (3D projections and horizontal) with G) lateral ventricular volume quantification, n = 5. H) Representative ventriculo‐cisternal perfusion time course of the dextran ratio (outflow/inflow) in a control (AAV‐GFP) and an NKCC1‐KD (AAV‐Cre) mouse 3 weeks post‐AAV injection, with summarized CSF production rates, obtained from the average of the time points indicated in a dashed box, n  =  6 of each. I) ICP measurements in control and NKCC1‐KD mice, 3 weeks post‐AAV injection, n  = 8 control and n = 6 NKCC1‐KD. Statistical evaluation with one‐way ANOVA with Sidak's multiple comparison post hoc test (panel B) or Student's t‐test (remaining panels). ^**^; *P* < 0.01, ^***^; *P* < 0.001, ns; not significant.

To determine the quantitative contribution of choroid plexus NKCC1 to CSF secretion without potential off‐target effects of pharmacological NKCC1 inhibitors, we employed the same AAV‐Cre‐based approach to knock down NKCC1 in choroid plexus of SLC12A2^flox/flox^ mice, henceforth termed “NKCC1‐KD” (**Figure** **3**A), with AAV‐GFP serving as control. The NKCC1‐coding transcript was significantly reduced already one week after virus injection (78% after one week, 77% after two weeks, 80% after three weeks, n = 3 for 1–2 weeks and n = 5 for 3 weeks, *P* < 0.001, Figure 3B) with parallel qPCR of transcripts encoding other transporters involved in CSF secretion (*ATP1A1*, *SLC4A2*, *SLC4A5*, *SLC4A10*) demonstrating no significant reduction in expression levels (Figure S3A–D, Supporting Information). The protein expression of NKCC1 was robustly reduced in choroid plexus following the AAV injection, as visualized with its near absence in immunohistochemical staining (n = 3 mice, see representative micrograph in Figure 3C) and quantified with Western blot (Figure 3D), which demonstrated a 69 ± 6% reduction of NKCC1 in choroid plexus from NKCC1‐KD mice (AAV‐Cre‐injected SLC12A2^flox/flox^ mice) as compared to choroid plexus from control mice (AAV‐GFP‐injected SLC12A2^flox/flox^ mice) three weeks post‐injection (n = 3 independent experiments with 3–5 biological replicates, see Figure 3D for representative experiments and Figure S3E (Supporting Information) for all Western blots). Electron microscopy of choroid plexus from the NKCC1‐KD mice demonstrated no morphological or structural difference from that obtained from control mice, Figure 3E, and thus no indication of altered cell volume or changes intercellular spaces. However, infiltrating migrating cells were observed in the choroid plexus stroma in some of the NKCC1‐KD mice, an example of which is illustrated in Figure S3F (Supporting information). The NKCC1‐KD mice displayed a robust ≈65% decrease in lateral ventricle volume (2.21 ± 0.17 mm^3^, n = 5 in NKCC1‐KD mice versus 6.22 ± 0.28 mm^3^, n = 5 in control mice, *P* < 0.001, Figure 3F,G (see Figure S3G–I,Supporting Information for quantification of other CSF spaces). The CSF secretion rate was significantly reduced by ≈40% in NKCC1‐KD mice (0.52 ± 0.03 µL min^−1^, n = 6) as compared to control mice (0.85 ± 0.06 µL min^−1^, n = 6, *P* < 0.001, Figure 3H), which is similar to the 45% reduction of the CSF secretion rate we previously demonstrated in WT mice upon intraventricular delivery of the NKCC1 inhibitor bumetanide.^[^ ^35^ ^]^ Accordingly, the NKCC1‐KD mice demonstrated reduced ICP (2.80 ± 0.30 mmHg, n = 6) compared to the control mice (4.19 ± 0.35 mmHg, n = 8, *P* < 0.05, Figure 3I). Taken together, these data demonstrate a key role for NKCC1 in CSF secretion and thus ventricular volume and ICP.

### 2.7 Elevation of [K^+^]_CSF_ Modulates NKCC1 and Na^+^/K^+^‐ATPase Activity While Maintaining Stable CSF Secretion

**Figure 4:**
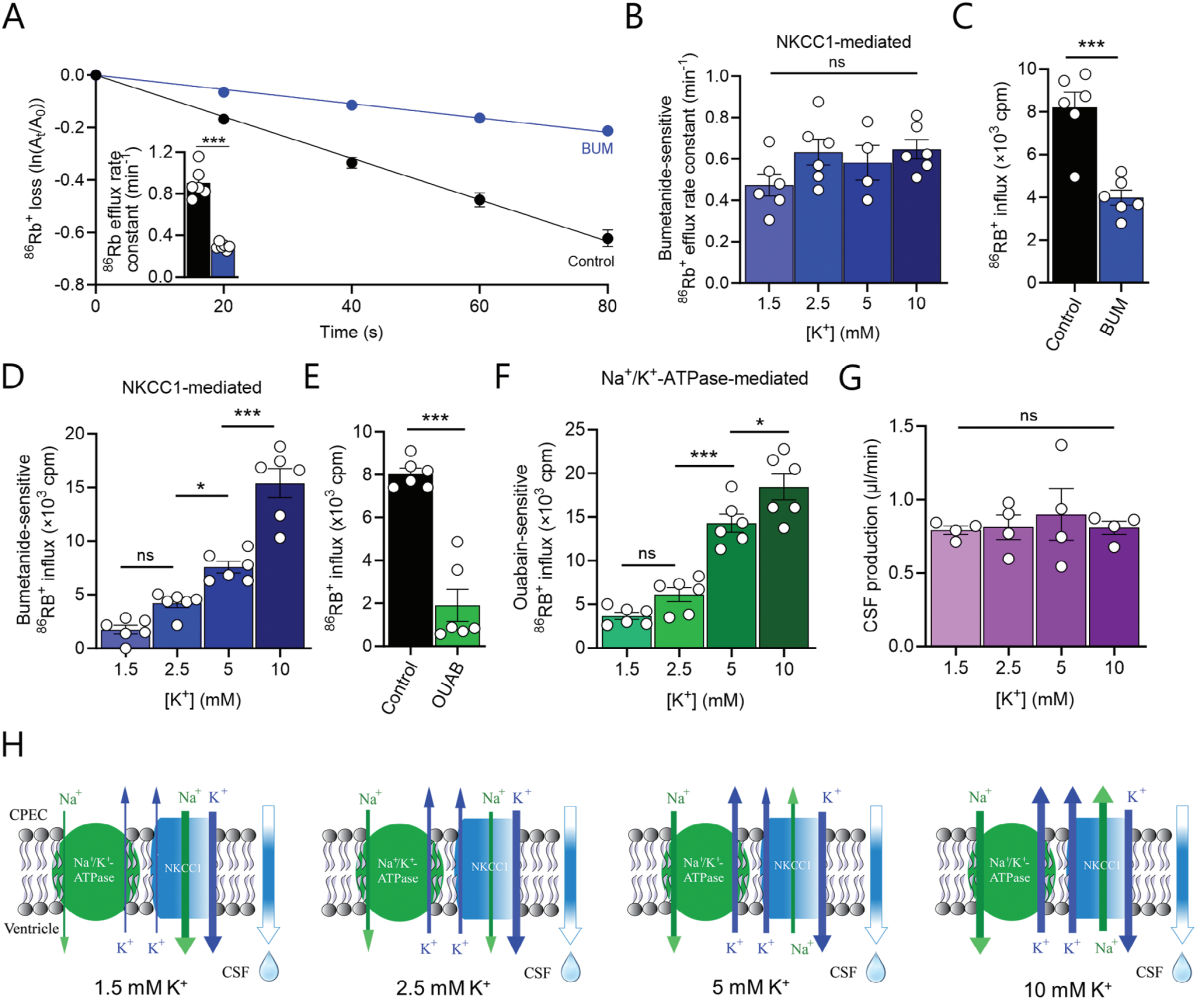
Elevation of [K^+^]_CSF_ modulates NKCC1 and Na^+^/K^+^‐ATPase activity while maintaining stable CSF secretion. A) Efflux of ^86^Rb^+^ from mouse choroid plexus in control aCSF (2.5 mm K^+^) without or with NKCC1 inhibition by bumetanide (BUM), n = 6 of each with efflux rate constants for ^86^Rb^+^ in insert. B) Bumetanide‐sensitive (NKCC1‐mediated) efflux rate constants for ^86^Rb^+^ with increasing [K^+^], n = 4 for 5 mm and n = 6 for remaining groups. C) Influx of ^86^Rb^+^ from mouse choroid plexus in control aCSF (2.5 mm K^+^) without or with NKCC1 inhibition by bumetanide (BUM), n = 6. D) Bumetanide‐sensitive (NKCC1‐mediated) influx of ^86^Rb^+^ with increasing [K^+^], n = 6 of each. E) Influx of ^86^Rb^+^ in mouse choroid plexus in control aCSF (2.5 mm K^+^) without or with Na^+^/K^+^‐ATPase inhibition by ouabain (OUAB), n = 6 of each. F) Ouabain‐sensitive (Na^+^/K^+^‐ATPase‐mediated) influx of ^86^Rb^+^ with increasing [K^+^], n = 6 of each. G) Summarized CSF production rates in mice with increasing [K^+^] in the infusion solution (final ventricular [K^+^] of 1.5 – 10 mm), n = 4 of each. H) Schematic depicting K^+^‐induced reversal of NKCC1‐mediated net transport combined with the increased Na^+^/K^+^‐ATPase‐mediated transport, yielding constant CSF production rate. Statistical evaluation with Student's t‐test (Panels A,C,E) or one‐way ANOVA with Tukey's post hoc test (Panels B,D,F,G). ^*^; *P* < 0.05, ^***^; *P* < 0.001, ns; not significant.

Pathological conditions may confer a rise in the CSF K^+^ concentration ([K^+^]_CSF_), which may modulate K^+^‐transporters in the choroid plexus and thus the CSF secretion rate.^[^ ^37^, ^38^, ^40^, ^41^, ^48^ ^]^ To determine the effect of elevated [K^+^]_CSF_ on the choroid plexus transporters NKCC1 and the Na^+^/K^+^‐ATPase, and their interplay, we employed WT mice (C57BL/6J) to retain all transport activity intact and performed isotope flux assays on excised choroid plexus from these mice with ^86^Rb^+^ serving as a congener of the transported K^+^. The rate of ^86^Rb^+^ efflux from pre‐equilibrated choroid plexus was reduced by ≈70% by inclusion of the NKCC1 inhibitor bumetanide (from 0.91 min^−1^ ± 0.06 in control aCSF containing 2.5 mm K^+^ to 0.30 min^−1^ ± 0.01 upon inclusion of bumetanide, n = 6 of each, *P* < 0.001, **Figure** **4**A), the difference of which representing the NKCC1‐mediated K^+^ efflux from choroid plexus. NKCC1‐mediated efflux (the bumetanide‐sensitive ^86^Rb^+^ efflux rate constant) was unaffected by the [K^+^] in the surrounding aCSF (in min^−1^: rates of 0.47 ± 0.05 in 1.5 mm K^+^, 0.63 ± 0.06 in 2.5 mm K^+^, 0.58 ± 0.08 in 5 mm K^+^, and 0.65 ± 0.05 in 10 mm K^+^, n = 4 for 5 mm and n = 6 for the remaining groups, *P* = 0.17, Figure 4B, see Figure S4A,B (Supporting Information) for the individual graphs obtained in control solution and in the presence of bumetanide prior to their deduction). As NKCC1 transports its substrates in both directions, with the net transport rate dictated by the ion concentrations on both sides of the membrane according to the Gibbs free energy equation, we determined the NKCC1‐mediated K^+^ uptake with a ^86^Rb^+^ influx assay. The rate of ^86^Rb^+^ uptake in ex vivo choroid plexus exposed to aCSF containing 2.5 mm K^+^ was reduced by ≈50% by inclusion of the NKCC1 inhibitor bumetanide (from 8.20 ± 0.71 × 10^3^ cpm to 3.99 ± 0.35 × 10^3^ cpm with bumetanide, n = 6 of each, *P* < 0.001, Figure 4C), the difference of which representing the NKCC1‐mediated K^+^ influx to the choroid plexus. The NKCC1‐mediated K^+^ uptake (the bumetanide‐sensitive ^86^Rb^+^ influx) appeared lower upon reduction of the [K^+^] in the surrounding aCSF from the basal 2.5 to 1.5 mm (in × 10^3^ cpm: 4.21 ± 0.42 in 2.5 mm K^+^ versus 1.75 ± 0.42 in 1.5 mm K^+^, n = 6 of each), although the difference did not reach statistical significance (*P* = 0.15), Figure 4D. Increasing [K^+^]_CSF_ promoted elevated NKCC1‐mediated K^+^ uptake (in ×10^3^ cpm: 4.21 ± 0.42 in 2.5 mm K^+^ versus 7.57 ± 0.54 in 5 mm K^+^, n = 6 of each, *P* < 0.05), and further in 10 mm K^+^ (in ×10^3^ cpm: 15.4 ± 1.35, n = 6 of each, *P* < 0.001, Figure 4D, see Figure S4C,D (Supporting Information) for the individual graphs obtained in control solution and in the presence of bumetanide prior to their deduction). Increased [K^+^]_CSF_ thus promotes an increase in the inwardly‐directed NKCC1 transport.

The transport activity of the neighboring K^+^ uptake mechanism, the Na^+^/K^+^‐ATPase, also abundantly expressed in the choroid plexus luminal membrane, was resolved with the ^86^Rb^+^ uptake assay in excised choroid plexus. The rate of ^86^Rb^+^ influx in ex vivo choroid plexus exposed to aCSF containing 2.5 mm K^+^ was reduced by ∼80% by inclusion of the Na^+^/K^+^‐ATPase inhibitor ouabain (from 8.03 ± 0.27 × 10^3^ cpm to 1.90 ± 0.75 × 10^3^ cpm with ouabain, n = 6 of each, *P* < 0.001, Figure 4E), the difference of which representing the Na^+^/K^+^‐ATPase‐mediated K^+^ influx to the choroid plexus. The Na^+^/K^+^‐ATPase‐mediated K^+^ uptake (the ouabain‐sensitive ^86^Rb^+^ influx) appeared lower upon reduction of the [K^+^] in the surrounding aCSF from the basal 2.5 to 1.5 mm (in × 10^3^ cpm: 6.13 ± 0.80 in 2.5 mm K^+^ versus 3.68 ± 0.41 in 1.5 mm K^+^, n = 6 of each), although the difference did not reach statistical significance (*P* = 0.34, Figure 4F). Increasing [K^+^]_CSF_ promoted elevated Na^+^/K^+^‐ATPase‐mediated K^+^ uptake (in × 10^3^ cpm: 6.13 ± 0.80 in 2.5 mm K^+^versus 14.29 ± 1.04 in 5 mm K^+^, n = 6 of each, *P* < 0.001), and further in 10 mm K^+^ (in × 10^3^ cpm: 18.45 ± 1.48, n = 6 of each, *P* < 0.05, Figure 4F, see Figure S4E,F (Supporting Information) for the individual graphs obtained in control solution and in the presence of ouabain prior to their deduction).

Elevation of the [K^+^]_CSF_ is thus expected to shift NKCC1 net transport direction inward, thus *lowerin*g its predicted net contribution to CSF secretion, while the Na^+^/K^+^‐ATPase responds to elevated [K^+^]_CSF_ with increased transport activity and thus an *increased* net contribution to CSF secretion. To determine the combined effect of modulation of [K^+^]_CSF_ in vivo, we determined the rate of CSF secretion in WT mice (C57BL/6J) by ventriculo‐cisternal perfusion with the increased [K^+^]_CSF_ in the ventricular perfusate. The CSF secretion rate was undisturbed by the varying [K^+^]_CSF_ (in µL min^−1^: 0.79 ± 0.03 in 1.5 mm K^+^, 0.81 ± 0.08 in 2.5 mm K^+^, 0.90 ± 0.18 in 5 mm K^+^, and 0.81 ± 0.05 in 10 mm K^+^, n = 4 of each, *P* = 0.87, Figure 4G). These data suggest that the K^+^‐induced *reduction* of NKCC1‐mediated outward electrolyte transport combined with the *increased* Na^+^/K^+^‐ATPase‐mediated transport serve to reduce the CSF [K^+^] load, while simultaneously balancing the CSF secretion rate in situations with elevated CSF [K^+^] (Figure 4H).

### 2.8 Subarachnoid Hemorrhage Does Not Elevate [K^+^]_CSF_ in Patients

**Figure 5:**
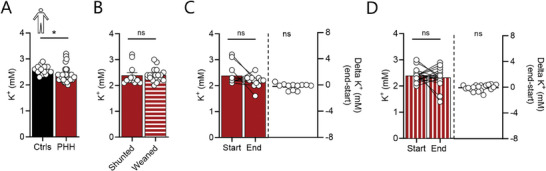
Patients with subarachnoid hemorrhage display undisturbed [K^+^]_CSF_. A) [K^+^] in CSF sampled upon placement and removal of an EVD from patients with posthemorrhagic hemorrhage (PHH) following subarachnoid hemorrhage, n = 32 and during preventive clipping of an unruptured aneurysm (Ctrls), n = 14. B) [K^+^] in CSF from shunted patients with subarachnoid hemorrhage (n = 12) versus those successfully weaned from the EVD (n = 20). C) [K^+^]_CSF_ in start samples (upon EVD placement) and end samples (upon removal of EVD) in CSF from shunted patients, with the delta [K^+^]_CSF_ on the right (n = 12). D) [K^+^]_CSF_ in start samples (upon EVD placement) and end samples (upon removal of EVD) in CSF from weaned patients, with the delta [K^+^]_CSF_ on the right (n = 19). Statistical evaluation with Student's t‐test or one sample t‐test (delta [K^+^]_CSF_). ^*^; *P* < 0.05, ns; not significant.

To determine if the [K^+^]_CSF_ is altered in patients suffering from PHH following subarachnoid hemorrhage, we quantified the [K^+^] in CSF samples obtained from the ventricular compartment upon placement of an extraventricular drain at hospital admission, in comparison to control cisternal CSF samples obtained from patients undergoing preventive vascular clipping of an un‐ruptured aneurysm. The [K^+^]_CSF_ in PHH patients was slightly *lower* than that obtained from control subjects (compare 2.38 ± 0.05 mm K^+^ in PHH, n = 32 with 2.58 ± 0.04 mm K^+^ in control, n = 14, *P* < 0.05, **Figure** **5**A). The [K^+^]_CSF_ did not differ between the group of patients that developed chronic hydrocephalus, and therefore had ventricular shunt placement (2.38 ± 0.11, n = 12), and those that did not, and therefore were successfully weaned off their extraventricular drain (2.40 ± 0.05, n = 20, *P* = 0.92, Figure 5B). Lastly, we compared the [K^+^]_CSF_ in the start samples and in the end samples (the latter obtained upon removal of the extraventricular drain, see Experimental Section) to determine whether a time‐dependent change in [K^+^]_CSF_ could account for the formation of chronic hydrocephalus. The [K^+^]_CSF_ was not significantly reduced from the time of the initial sampling to the time of end sampling in the shunted patients (0.22 ± 0.13 mm reduction, n = 12, *P* = 0.13, Figure 5C) or in those weaned from their extraventricular drain (0.08 ± 0.11 mm reduction, n = 19, *P* = 0.47, Figure 5D). Thus, the stable [K^+^]_CSF_ across the control and patient CSF, and during the hydrocephalus formation, suggests that the [K^+^]_CSF_ in patients with subarachnoid hemorrhage is not a molecular driver of acute or chronic PHH formation.

## 3 Discussion

We here demonstrate that the choroid plexus AQP1 is not required for CSF secretion or brain water homeostasis in mice and confirm that the cotransporter NKCC1 is a key contributor to CSF secretion in physiology. With its abundance and polarized expression in the luminal membrane of murine choroid plexus,^[^ ^22^, ^49^ ^]^ AQP1 conferred ≈60% of the osmotic water permeability across the luminal membrane of the excised choroid plexus, as determined with choroid plexus obtained from AQP1^−/−^ mice. These acutely excised choroid plexuses were exposed to an abrupt osmotic challenge, which extracts fluid across the luminal membrane, and thus solely illustrates the osmotic water permeability of the AQP1‐expressing membrane in the choroid plexus. An earlier study arrived at ≈80% reduction in the osmotic water permeability across the luminal membrane of AQP1^−/−^ choroid plexus.^[^ ^23^ ^]^ This discrepancy may originate from the former study's use of t_1/2_ analysis of the trace, rather than quantification of the initial rate of cell swelling, and dilution of the aCSF with distilled water, rather than maintaining the electrolyte content by addition and removal of mannitol.^[^ ^23^, ^50^ ^]^ The latter approach prevents alterations in ion concentrations and thus membrane potential and driving forces for the various membrane transporters involved in electrolyte and fluid transport. Irrespective of the large AQP1‐mediated osmotic water permeability across the luminal membrane, the transepithelial osmotic permeability of rat choroid plexus (L_p_ = 9 × 10^−5^ cm s^−1^Osm^−1^,^[^ ^9^, ^51^ ^]^) ranges amongst the epithelia with low osmotic water permeability^[^ ^4^ ^]^ due to the lack of AQPs in the basolateral membrane.^[^ ^22^ ^]^ Notably, there is no osmotic gradient across the choroid plexus in vivo (i.e., comparable plasma and CSF osmolarities,^[^ ^9^, ^27^, ^52^, ^53^ ^]^) and no inter‐microvillar or lacunar local osmotic compartment to drive the CSF flow.^[^ ^9^, ^54^ ^]^ In addition, the choroid plexus, and other epithelia,^[^ ^55^, ^56^, ^57^ ^]^ is able to secrete CSF independently, and even against, an osmotic gradient, as reported for goats, cats, rabbits, and rats.^[^ ^9^, ^27^, ^28^, ^51^ ^]^ Combined with reports that AQP1‐deficient humans exhibit no neurological deficits,^[^ ^25^, ^26^ ^]^ this ability to secrete CSF independently of a transepithelial bulk osmotic gradient suggests that CSF secretion may not solely rely on conventional AQP‐mediated osmotic water transport. Accordingly, in the in vivo experimentation, where no externally applied osmotic gradient is included, AQP1^−/−^ mice displayed no difference in CSF secretion rate, brain water content, ICP, or ventricle size. These findings conflict with the earlier study that demonstrated a modest (20%) reduction in CSF secretion rate in the AQP1^−/−^ mice and a reduced ICP,^[^ ^23^ ^]^ which could both be influenced by the lack of mechanical ventilation of the anesthetized mice and blood gas monitoring in the earlier study.^[^ ^23^ ^]^ Compromised physiological parameters reduce CSF secretion rates in mice,^[^ ^35^ ^]^ and may cause the 80% reduction in central venous pressure, which the authors assigned as a contributor to the observed ICP reduction.^[^ ^23^ ^]^ The AQP1^−/−^ mice had larger diuresis due to kidney AQP1 deficiency, which promotes an increased water intake (this study and ^[^ ^24^, ^43^, ^44^ ^]^). Consequently, we observed similar systemic water composition in AQP1^−/−^ mice and their WT littermates. To verify that systemic AQP1 deficiency did not indirectly influence the CSF secretion rate in AQP1‐deficient mice, we employed the Cre‐LOX system to selectively induce knockdown of AQP1 in choroid plexus of AQP1^flox/flox^ mice. Cre was introduced by stereotaxic intraventricular delivery of AAV5, which selectively targets the choroid plexus epithelium (this study and ^[^ ^45^ ^]^), and nearly abolished the AQP1 transcript abundance 1–3 weeks post‐delivery (with no significant modulation of transcripts encoding other transport proteins involved in CSF secretion). Three weeks post‐delivery, the AQP1 protein expression in choroid plexus was nearly undetectable with immunohistochemical staining and reduced by 60% in Western blotting, but undisturbed in kidney tissue. At this time point, mice with knockdown of AQP1 displayed similar CSF secretion rate, brain water content, and ventricular volume, as mice exposed to the control AAV. Taken together, AQP1 does not appear to be required for CSF secretion and brain fluid homeostasis in healthy mice. It remains unresolved whether this finding will extend to pathological conditions, where one could speculate that potential disease‐induced alterations in ventricular osmolarity could contribute to hydrocephalus formation. However, no changes in ventricular CSF osmolarity was observed in patients with subarachnoid hemorrhage and subsequent post‐hemorrhagic hydrocephalus in comparison to cisternal CSF obtained from patients undergoing preventive vascular clipping of an unruptured aneurysm.^[^ ^52^ ^]^ Nor was any such osmolarity increase detected in CSF (ventricular or lumbar) obtained from patients with normal pressure hydrocephalus.^[^ ^53^ ^]^ As AQP‐mediated osmotic water flow requires an osmotic gradient, we suspect a limited contribution of AQP1 to hydrocephalus formation in these pathologies.

NKCC1 is amongst the most highly expressed transporters in the choroid plexus of rodents, zebrafish, and humans^[^ ^49^ ^]^ and located on the luminal membrane.^[^ ^35^, ^58^ ^]^ Its ∼ 40% contribution to CSF secretion has been demonstrated in vivo with pharmacological inhibition of NKCC1 by intraventricular delivery of bumetanide in dogs, mice, and rats, with three different experimental approaches (the ventriculo‐perfusion assay, the LI‐COR‐based CSF live imaging, and the so‐called “direct approach”).^[^ ^9^, ^32^, ^33^, ^35^ ^]^ We here cement choroid plexus NKCC1 as a key contributor to CSF secretion. Intraventricular delivery of Cre‐containing AAV5 in SLC12A2^flox/flox^mice caused an 80% reduction in choroid plexus SLC12A2 transcript 1–3 weeks post‐delivery, near‐absence of NKCC1 in immunohistochemical staining, and a 70% reduction in NKCC1 protein expression determined by Western blotting three weeks post‐delivery. We observed a statistical tendency, but no significance, toward a reduction in transcripts encoding other transport proteins involved in CSF secretion, which could potentially arise as a compensatory mechanism following such robust knockdown of NKCC1, but also contribute slightly to the reduced rate of CSF secretion in the Cre‐treated animals. At three weeks, the choroid plexus ultrastructural morphology and apparent epithelial cell volume were intact, but the CSF secretion rate reduced by 40%, the ventricle size by 60%, and the ICP by 35%. These data confirm NKCC1 as key contributor to choroid plexus‐mediated CSF secretion, at least in part by its ability to conduct cotransporter‐mediated water transport. NKCC1 and other cotransporters have been demonstrated to possess an ability to transport water in the direction of its transported solutes in a manner inherent in the protein itself,^[^ ^59^, ^60^ ^]^ for reviews, see.^[^ ^34^, ^61^ ^]^ This mode of water transport allows a fixed number of water molecules to be translocated across the membrane with each transporter turnover, independently of – and even against – the prevailing transmembrane osmotic gradient.^[^ ^4^, ^62^ ^]^ We have previously demonstrated such NKCC1‐*dependent*, but transmembrane osmolarity‐*independent* water transport across the luminal membrane of acutely excised mouse and rat choroid plexus,^[^ ^9^, ^35^ ^]^ which will allow for NKCC1‐mediated CSF secretion in the absence of a transchoroidal osmotic gradient^[^ ^9^ ^]^ and/or functional AQP1 expression (this study).

NKCC1 readily transports its solutes bi‐directionally, with its net transport direction dictated by the substrate concentrations on either side of the plasma membrane, dictated by Gibbs free energy equation. This feature is shared amongst all coupled transporters. Our earlier quantification of the Na^+^, K^+^, and Cl^−^ gradients across the acutely excised murine choroid plexus predicted an outwardly‐directed net transport by NKCC1 at physiological ion concentrations,^[^ ^35^ ^]^ which was demonstrated experimentally with live [Na^+^]_i_ imaging of acutely excised murine and rat choroid plexus.^[^ ^9^, ^35^ ^]^ However, an elevation in [K^+^]_CSF_ could promote a change in net transport direction of NKCC1 toward its inward direction, and could as such reduce the NKCC1‐mediated contribution to CSF secretion. The brain is notoriously capable of sustaining a stable [K^+^] in the CSF and extracellular fluid despite sustained plasma hyperkalemia.^[^ ^63^, ^64^ ^]^ This strict regulation is mainly conferred by the Na^+^/K^+^‐ATPase located in the abluminal endothelial membrane and the luminal membrane of the choroid plexus.^[^ ^65^, ^66^ ^]^ The Na^+^/K^+^‐ATPase isoform combination expressed in these brain barrier cells provides the pump with kinetic characteristics that allow the pump to increase its activity with elevated extracellular [K^+^].^[^ ^67^, ^68^ ^]^ A smaller additional K^+^‐clearance component, previously undefined,^[^ ^68^ ^]^ may well be assigned to NKCC1 (for review, see^[^ ^69^ ^]^). Accordingly, we here demonstrate that choroid plexus Na^+^/K^+^‐ATPase activity increased robustly upon an increase in the surrounding [K^+^], with a 230% increased activity by doubling of the [K^+^]_CSF_ from 2.5 to 5 mm. The NKCC1‐mediated K^+^ *efflux* from the choroid plexus was undisturbed by altered [K^+^] in the surrounding fluid, while the NKCC1‐mediated *influx* increased with an increase in the surrounding [K^+^], with a 70% increase by doubling of the [K^+^]_CSF_. At high [K^+^]_CSF_, the NKCC1 net transport direction will thus be inward, but the Na^+^/K^+^‐ATPase activity, and thus Na^+^ transport from the choroid plexus to the ventricle, will increase, both of which will contribute to the desired clearance of the elevated [K^+^]_CSF_ from the ventricular lumen. Notably, with these altered transport rates, the Na^+^/K^+^‐ATPase‐mediated contribution to CSF secretion will *increase* and the NKCC1‐mediated contribution to CSF secretion *decrease*. The combined outcome of which was here demonstrated to result in a stable rate of CSF secretion across the tested [K^+^]_CSF_ from 1.5 to 10 mm. In this manner, when faced with elevated brain [K^+^], the choroid plexus transport mechanisms NKCC1 and Na^+^/K^+^‐ATPase work in concert to restore the [K^+^]_CSF_ to basal levels by clearing K^+^ from the ventricles while retaining the CSF secretion rate constant.

It has been proposed that brain hemorrhage may result in elevated ventricular [K^+^]_CSF_,^[^ ^40^ ^]^ which could promote such reversal of NKCC1. However, we here demonstrate that the [K^+^] in CSF obtained during extraventricular drain placement in patients with subarachnoid hemorrhage was similar, or even slightly reduced, compared to cisternal CSF obtained from healthy individuals undergoing preventive vascular clipping of an un‐ruptured aneurysm. This stable [K^+^]_CSF_ suggests that NKCC1 will remain in its net outwardly‐transporting mode during post‐hemorrhagic hydrocephalus. The [K^+^]_CSF_ of ∼2.5 mm aligns with that obtained by Sadegh and colleagues in CSF from patients with subarachnoid hemorrhage,^[^ ^40^ ^]^ and did not differ amongst patients developing chronic hydrocephalus and requiring permanent shunt placement versus those that were successfully weaned of the temporary extraventricular drain. [K^+^]_CSF_ did not change in any of the patient groups from the time of extraventricular drain placement to the time of its removal, although we cannot rule out that fluctuations occurred in the interval between the two time points or that putative osmotherapy prior to external drain placement could affect the [K^+^]_CSF_. However, earlier analysis of these CSF samples from patients with subarachnoid hemorrhage illustrated osmolarity and electrolyte content identical to samples obtained from otherwise healthy patients undergoing preventive vascular clipping of an unruptured aneurysm,^[^ ^52^ ^]^ supporting a [K^+^]_CSF_ within the physiological range in patients with PHH. Taken together, such lack of brain hemorrhage‐induced increase in [K^+^]_CSF_ is at odds with the recently proposed K^+^‐induced NKCC1‐mediated reversal, promoting choroid plexus as a site of CSF reabsorption following brain hemorrhage.^[^ ^40^ ^]^ Rather, the stable [K^+^]_CSF_ obtained in the present patient cohort suggests that NKCC1 retains its net outward transport direction and its continued contribution to CSF secretion during a hemorrhagic event. These data align with previously published demonstrations of NKCC1‐mediated *increase* in brain fluid content and ventriculomegaly in rat models of subarachnoid hemorrhage, intraventricular hemorrhage, and traumatic brain injury,^[^ ^33^, ^70^, ^71^ ^]^ and the associated improved outcome upon NKCC1 inhibition.^[^ ^40^ ^]^

### 3.1 Limitations

A genetic approach to transporter elimination, as here employed, provides certain advantages over pharmacologically‐induced inhibition of select transport proteins, but also a limitation in a sense that each of these knockout or knockdown approaches require a different mouse strain (the one in which the AQP1^−/−^ mice were originally generated (Figure 1), the two different floxed strains (AQP1^flox/flox^ and SLC12A2^flox/flox^, Figures 2 and 3), in addition to a generic wildtype for K^+^‐dependent transporter function (Figure 4), see Experimental Section for details). These strains display different CSF secretion rates and different ventricular sizes, the latter of which also observed by others.^[^ ^72^, ^73^ ^]^ Similarly, we have earlier observed strain‐specific CSF secretion rates and ventricular sizes in rats (Sprague Dawley, Wistar, Zucker, and spontaneous hypertensive rats (SHR)),^[^ ^74^, ^75^, ^76^, ^77^ ^]^ which, together, indicate biological strain‐specific differences in these parameters. We therefore took great care to employ the parental strain littermates as control for each experimental series. Nevertheless, we cannot rule out that any of these, although small, differences may affect the data acquisition in unknown ways. However, the AQP1 KO/KD mice had similar ventricular sizes as their control counterparts, which should provide similar conditions for the ventriculo‐cisternal perfusion assay. The NKCC1‐KD mice had significantly smaller ventricles (due to the lower CSF secretion rates). This parameter could represent a confounding element in the experimental approach, although we have in a previous study obtained the same reduction in CSF secretion rate upon acute pharmacological inhibition of NKCC1 (and thus similar ventricle size),^[^ ^35^ ^]^ suggesting that abolishing NKCC1 transport activity – whether genetically or pharmacologically – reduces the CSF secretion rate by ≈40%.

## 4 Conclusion

In conclusion, we here demonstrate that choroid plexus AQP1 is not required for CSF secretion under physiological conditions, which aligns with the lack of neurological symptoms in patients deficient in AQP1 protein expression and with the reported ability of choroid plexus‐mediated CSF secretion to take place against an osmotic gradient. The NKCC1‐mediated contribution to CSF secretion was cemented with choroid plexus‐specific knockdown of NKCC1 yielding identical results as those obtained with intraventricular delivery of an NKCC1 inhibitor, as was its ability to revert its net transport direction with elevated [K^+^] in the CSF. The combined effect on NKCC1 and the Na^+^/K^+^‐ATPase of elevated [K^+^] promotes clearance of a ventricular [K^+^]_CSF_ load while maintaining a stable rate of CSF secretion. This dual‐facetted function is considered a physiological advantage. Finally, we failed to observe increased [K^+^]_CSF_ in patients with subarachnoid hemorrhage and thus propose that NKCC1 retains its net outward transport rate and its associated contribution to development of post‐hemorrhagic hydrocephalus. Our data thus support a future modulation of NKCC1, indirectly^[^ ^75^, ^78^ ^]^ or directly,^[^ ^33^, ^71^ ^]^ as a mode of pharmacological modulation of the ventriculomegaly often observed in patients following a hemorrhagic event.

## 5 Experimental Section

### Animals

All animal procedures conformed to European guidelines (EU Directive 2010/63/EU) and were approved by the Danish Animal Experiments Inspectorate (permission no. 2018‐15‐0201‐01595, 2019‐15‐0201‐01629, 2021‐15‐0201‐00867, and 2024‐15‐0201‐00867). Experiments were performed on male wild‐type (WT) (C57BL/6J, Janvier), AQP1 knockout (AQP1^−/−^, originally generated in CD1 mice),^[^ ^24^ ^]^ AQP1^flox/flox^ mice (originally generated in C57BL6 mice)^[^ ^79^ ^]^ (courtesy of Professor Andreas Wagner, Heidelberg University), and NKCC1^flox/flox^mice (originally generated in C57BL6 mice).^[^ ^80^ ^]^ Both lines of floxed mice were bred as homozygous floxed mice (^flox/flox^), whereas the AQP1^−/−^ mice were generated by mating AQP1^+/‐^mice, and subsequently employing the AQP1^−/−^ and the littermate WT offspring for experiments. Mice were housed with a 12:12 light cycle and with access to food and water ad libitum. AQP1^−/−^were used at 10–11 weeks of age (with exception of water intake/urinary volume experiments, where the mice were 12 and 19 weeks of age), and AQP1^flox/flox^ and NKCC1^flox/flox^ mice were injected with a virus at 8–10 weeks of age with further procedures performed 3–4 weeks later unless otherwise stated.

### Solutions

Experiments were performed with CO_2_/HCO_3_^−^‐buffered artificial cerebrospinal fluid (HCO_3_^−^‐aCSF; (in mm) 120 NaCl, 2.5 KCl, 2.5 CaCl_2_, 1.3 MgSO_4_, 1 NaH_2_PO_4_, 10 glucose, 25 NaHCO_3_, equilibrated with 95% O_2_/5% CO_2_ to obtain a pH of 7.4) when experimentally feasible. In experiments where the solution could not be equilibrated with 95% O_2_/5% CO_2_, aCSF was instead buffered by HEPES (HEPES‐aCSF; (in mm) 120 NaCl, 2.5 KCl, 2.5 CaCl_2_, 1.3 MgSO_4_, 1 NaH_2_PO_4_, 10 glucose, 17 Na‐HEPES, adjusted to pH 7.4 with NaOH). The HEPES‐aCSF was used for osmotic water permeability measurements and isotope tracer experiments, the latter of which with previous demonstration of similar ^86^Rb^+^ efflux rates with the two differentially‐buffered solutions.^[^ ^81^ ^]^

### Anesthesia and Ventilation

For all acute in vivo experiments, mice were anesthetized with xylazine (single initial intraperitoneal (i.p.) injection; 1 mg mL^−1^ and 0.1 mL per 10 g body weight, 37 °C, ScanVet), followed by an i.p. injection with ketamine (10 mg mL^−1^ and 0.1 mL per 10 g body weight plus 0.15 mL for mice <30 g and 0.20 mL for mice >30 g, 37 °C, MDS Animal Health). To sustain anesthesia, the mice were re‐dosed with half ketamine dose, as per detection of foot reflex. The body temperature was monitored and maintained at ≈37 °C using a homeothermic system (Harvard Apparatus). The mice were tracheostomized and mechanically ventilated (VentElite, Harvard Apparatus) with 0.9 L min^−1^ humidified air mixed with 0.1 L min^−1^ O_2_ and ventilation parameters were adjusted according to a pulse oximeter (MouseOx Plus, Starr Life Sciences) and capnograph readings (Type 340, Harvard Apparatus) to obtain physiological partial pressure of CO_2_ and arterial O_2_ saturation values (calibrated following blood gas quantification on an ABL800 FLEX, Radiometer).

### Total Body Water Content

Mice were weighed and body composition was measured by magnetic resonance imaging in unanesthetized mice (EchoMRI‐4in1Tm, Echo Medical system LLC, USA). The total body water content was calculated as a percentage of bodyweight.

### Water Intake and Urine Volume

Baseline measurements were conducted in 12‐week‐old AQP1^−/−^ and WT and 19‐week‐old AQP1^−/−^ and WT mice fed a standard rodent diet (TD.00374; Altromin Spezialfutter GmbH & Co. KG, Lage, Germany). The animals were housed in separate metabolic cages within a controlled temperature environment, with unrestricted access to food and drinking water. Measurements of food and water intake as well as urine output were recorded over 24 h after a period of acclimation lasting 3 days. On the day of measurement, the 19‐week‐old mice had their hydration switched to a non‐wetting water gel (HydroGel, Clear H20), whereas the 12‐week‐old mice retained their regular water bottle. The experiment was terminated by cervical dislocation of isoflurane‐anesthetized mice (Nicholas Piramal Ltd., London, UK).

### Osmotic Water Permeability

The acutely isolated lateral choroid plexus was stored in ice‐cold HEPES‐aCSF until experimentation (<3 h from isolation to end of the experiment). The choroid plexus was mounted on a coverslip treated with Corning Celltak (9 µL Cell‐Tak, 1 µL 2 m Na_2_CO_3_ and 0.33 µL isopropanol, dried, and rinsed with H_2_O), placed in a POCmini2 open perfusion chamber and loaded with calcein‐AM (17 µm in HEPES‐aCSF) for 10 minutes upon continuous perfusion with HEPES‐aCSF at a rate of ≈1 mL min^−1^. Live imaging was performed at 37 °C in an inverted microscope (Zeiss CellObserver with a Hamamatsu Orca Flash 4.0 LT detector, 20x/0.8 Plan Apochromat objective, 1 Hz acquisition rate). During the imaging, the perfusion solution was changed to HEPES‐aCSF containing an additional 100 mm mannitol. Analysis was performed using ImageJ (NIH). Images were converted to black/white (choroid plexus/background) using the Gaussian blur threshold, followed by placement of five regions of interest each containing 50% choroid plexus and 50% background. The change of distribution between choroid plexus and background was used to measure the relative change in choroid plexus area. This change was plotted as a function of time to obtain the initial linear part of the curve, with which to calculate the rate of osmotically‐induced change in choroid plexus area.

### Ventriculo‐Cisternal Perfusion

Mice were anesthetized and ventilated as described above. The mouse was placed in a stereotaxic frame (Harvard Apparatus) in the prone position and a midline incision was made over the skull and upper cervical spine. An infusion cannula (Brain infusion kit 3, Alzet) was placed 1.0 mm lateral to the midline (ML), 0.5 mm posterior to bregma (AP), and 2.5 mm ventral (DV) into the brain, and secured to the skull with dental resin cement (Panavia SA, Kuraray Noritake Dental Inc.). Using an inline heater (SF‐28, Warner Instruments), the perfusion solution was heated to 37 °C before entering the infusion cannula. A glass capillary (30–0018, Harvard Apparatus pulled by a Brown Micropipette puller, Model P‐97, Sutter Instruments) held in a 5° position was introduced into the cisterna magna. After observing CSF in the glass capillary, infusion of equilibrated (95% O_2_/5% CO_2_) aCSF (containing 0.5 mg mL^−1^ tetramethylrhodamine isothiocyanate‐dextran (TRITC‐dextran MW 155000, T1287 Sigma‐Aldrich) was initiated at a rate of 0.7‐0.8 µL min^−1^using a peristaltic pump, and outflow was collected in 5‐min intervals by introducing a second glass capillary into the fixed cisterna magna glass capillary. The fluorescence intensity was measured in a microplate photometer (545 nm, Synery Neo2 Multi‐mode Microplate reader, BioTek Instruments), and the average signal across the 60–75 minutes of infusion was used to calculate the CSF secretion rate with the formula:

(1) $$V_{p}=r_{i}\;\times \;\frac{C_{i}-C_{o}}{C_{o}}$$

where *V*_p_  =  CSF production rate (µL min^−1^), *r*_i_  =  infusion rate (µL min^−1^), *C*_i_  =  fluorescence of inflow solution, *C*_o_  =  fluorescence of outflow solution. In a set of experiments, the [K^+^] in the infused aCSF was changed (0.5, 7.5, and 17.5 mm). This aCSF will be diluted ≈1:1 with the endogenously secreted CSF (containing ∼ 2.5 mm K^+^) to a final estimated [K^+^]_CSF_ of 1.5, 5, and 10 mm). To ensure equiosmolarity, the [K^+^]_aCSF_ was obtained with equimolar exchange with Na^+^.

### Brain Water Content

Mice were euthanized by cervical dislocation and brains were quickly dissected into a pre‐weighed porcelain beaker (Witeg), weighed, and then kept at 100 °C for 72 h. The weight of the dehydrated brain tissue was employed to obtain the wet‐dry ratio and thus the brain water content in percentage.

### Magnetic Resonance Imaging (MRI)

Anesthetized mice underwent MRI in a 9.4 Tesla preclinical horizontal bore scanner (BioSpec 94/30 USR, Bruker BioSpin, Ettlingen, Germany) equipped with a 240 mT m^−1^ gradient coil (BGA‐12S, Bruker) at the Preclinical MRI Core Facility, University of Copenhagen. The scanner was interfaced to a Bruker Avance III console and controlled by Paravision 6.1 software (Bruker). Imaging was performed with a cryogenically‐cooled Tx/Rx quadrature‐resonator (CryoProbe, Bruker BioSpin). Body temperature was maintained at 37 ± 0.5 °C with a thermostatically controlled waterbed and the respiratory rate was monitored by an MR‐compatible monitoring system (SA Instruments, NY, USA). During the scan, anesthesia was maintained at 1‐1.5% isoflurane in a 1/1 mixture of air/oxygen. For obtaining high‐resolution CSF volumetry, a 3D constructive interference steady‐state sequence (3D‐CISS) image^[^ ^82^ ^]^ was calculated as a maximum intensity projection (MIP) from 4 realigned 3D‐TrueFISP volumes with 4 orthogonal phase encoding directions (TR = 4.2 ms, TE = 2.1 ms, NA = 1, Flip angle = 50°, 3D spatial resolution 75 × 75 × 75 µm, RF phase advance 0, 180, 90, 270°, TA = 14 minutes). To obtain optimal spatial uniformity, all acquired 3D‐TrueFISP volumes were motion‐corrected before calculation as MIP, and the image bias field was removed with Advanced Normalization Tools (ANTs).^[^ ^83^, ^84^ ^]^ For each brain sample, total brain volume was automatically segmented using region growing with ITK‐snap (version 3.8.0).^[^ ^85^ ^]^ In addition, pixel intensity factorized semi‐automatic thresholding and ROI segmentation were performed with Imalytics Preclinical software (ver.3.1.1.0, Gremse‐IT GmbH, Aachen, Germany).^[^ ^86^ ^]^ Volume measurements for both the whole brain and the segmented ventricular compartments were conducted in a blinded fashion using Imalytics Preclinical software.

### Intracranial Pressure Measurements

Mice were anesthetized and ventilated as described above, and an infusion cannula was placed as described in the section on ventriculo‐cisternal perfusion. The cannula was pre‐filled with HEPES‐aCSF and connected to a pressure transducer (APT300) and an amplifier module (TAM‐A, Hugo Sachs Elektronik). A small amount of HEPES‐aCSF was infused to secure a continuous fluid column between the pressure transducer and lateral ventricle, after which the ICP stabilized within 20 min. The average ICP value across the subsequent 5–10 min period was employed for quantification. The pressure signal was visualized and acquired at 1 kHz using the Basic Data Acquisition Software (BDAS, Havard Apparatus, Hugo Sachs Elektronik). Data was excluded if the ICP probe did not seal properly (one mouse in Figure 3I, group NKCC1‐KD).

### Viral Vector Injection

Surgeries were performed on isoflurane anaesthetized mice (4‐5% induction, 1.5‐2% maintenance) under aseptic conditions. Pre‐operatively, mice were administered analgesics subcutaneously (buprenorphine 0.05 mg kg^−1^, carprofen 5 mg kg^−1^). Prior to injection of viral vectors, mice were positioned in a stereotaxic frame (Harvard Apparatus) and a midline incision was performed to expose the skull. Each mouse received a single injection of 3.5 µL (1.3 × 10^13^ vg mL^−1^) of either AAV‐Cre (pAAV.CMV.HI.eGFP‐Cre.WPRE.SV40, Addgene viral prep #105545‐AAV5) or AAV‐GFP (pAAV.CMV.PI.EGFP.WPRE.bGH, Addgene viral prep #105530‐AAV5), Addgene, gift from James M. Wilson) into a lateral ventricle (0.5 mm posterior (AP) and 1.0 mm lateral to bregma (ML), 2.5 mm deep (DV), 1 µL s^−1^) using a Hamilton syringe. The needle was kept in place for 5 minutes to minimize backflow, before the incision site was closed with sutures (5‐0 Ethilon, 661H). Mice were treated with carprofen for 72 h (5 mg kg^−1^ every 24 h) and single‐housed after the procedure.

### Immunoblotting

Mice were euthanized by cervical dislocation and the brain quickly removed and placed in ice‐cold HEPES‐aCSF. The choroid plexus from the two lateral and the 4^th^ ventricle were isolated and kept on ‐80 °C until further use. The choroid plexuses were sonicated on ice (Sonoplus, Bandelin) in PBS or RIPA‐buffer with protease inhibitors. Western blotting was performed using precast SDS‐PAGE gels (mini‐PROTEAN, Biorad) and immobilon FL‐membranes (Sigma). Primary and secondary antibodies were diluted in a 1:1 mix of Odyssey blocking buffer (LI‐COR Biosciences) and PBS‐T. Primary antibodies: chicken anti‐GAPDH (AB2302, Sigma 1:900), anti‐GFP (Ab290, Abcam 1:5000), anti‐AQP1 (kindly provided by Robert Fenton 1:1000) and sheep anti‐NKCC1 (S022D, MRC, University of Dundee 1:250). Secondary antibodies: IRDye 680RD donkey anti‐chicken (Licor 670CW red, 926–68075), goat anti‐rabbit (Licor 800CW green, 906–32211), and rabbit anti‐sheep (Thermo Scientific 800CW green, SAS‐10060). Signals were detected with the Odyssey CLx imaging system and image analysis was performed with Image Studio 5.

### Immunohistochemistry

Mice were perfused with 0.9% saline followed by 4% paraformaldehyde in phosphate buffered saline (PBS, pH 7.4). The brain was removed and post‐fixed overnight in the same fixative, cryoprotected in 30% sucrose for ≥ 24 h, and frozen in crushed solid CO_2_. The brain was embedded in tissue freezing medium (Leica) and cut into 12 µm sections on a cryostat. Sections were washed in PBS and PBS‐T (0.3% Triton‐X), blocked for 3 h with 5% goat serum (G9023, Sigma) and incubated in primary antibodies overnight (mouse anti‐AQP1, 1:100, Santa Cruz, sc‐32737, rabbit anti‐NKCC1, 1:400, Abcam, ab59791). Sections were incubated in secondary antibodies for 2 h (goat anti‐rabbit 546, 1:500, Invitrogen A11010, goat anti‐mouse IgG2b 647, 1:500, Invitrogen A21242), washed and coverslipped with ProLong Gold Antifade mounting medium with DAPI (Thermofisher P36941). Images were acquired using an LSM 700 confocal microscope or with a Zeiss Axioscan.Z1 slide scanner with a 20 × /0.8 Plan Apochromat objective.

### Electron Microscopy

Mice were perfused via the left ventricle of the heart with Ringer's solution followed by Ringer's solution with 4% paraformaldehyde and 2% glutaraldehyde. The brain was removed and post‐fixed in the same fixative overnight, and subsequently stored in cacodylate buffer (Na‐cacodylate: 155 mm, CaCl: 1.1 m, adjusted to pH 7.4 with HCl) at 4 °C. The excised choroid plexuses were post‐fixed with 2% (w/v) osmiumtetroxide for 1 h at room temperature. During the following dehydration in ascending ethanol series, post‐staining with 1% (w/v) uranylacetate was performed. Afterward, the samples were embedded in epoxy resin (Araldite) and sectioned (thickness 70 nm) using a Leica Ultracut S (Leica, Wetzlar, Germany). Finally, the ultrathin sections were mounted on filmed Cu grids, post‐stained with lead citrate, and studied in a transmission electron microscope (EM 900, Zeiss, Oberkochen, Germany) at 80 kV and a magnification of 7,000x. For image recording, a 2K slow scan CCD camera (TRS, Moorenweis, Germany) was used.

### mRNA Quantification

The choroid plexus was dissected and kept in RNAlater (R0901, Sigma) at ‐80 °C. Total RNA was purified with the RNeasy micro kit (Qiagen) according to the manufacturer's instructions. Extracted RNA was quantified spectrophotometrically (Simplinano, Biochrom) and 50 ng of RNA was used for reverse transcription using the QuantiTect Reverse Transcription Kit (Qiagen) according to the manufacturer's instructions. RT‐qPCR was performed in triplicates in a QuantStudio7 Flex (Applied Biosystems) with TaqMan Gene Expression Master Mix (Applied Biosystems) and *Gapdh* as an internal control. Primers were purchased (Applied Biosystems) with the following catalog numbers: *Gapdh*: 4 331 182, *Slc12a2*: 4 331 182, *Aqp1*: 4 331 182, *Atp1a1*: 4 331 182, *Slc4a2*: 4 331 182, *Slc4a10*: 4 331 182, *Slc4a5*: 4 331 182. Analysis was performed with the 2^−ΔΔCT^method.

### ^86^Rb^+^ Tracer Flux Assays

The lateral choroid plexus was isolated and placed in ice‐cold HEPES‐aCSF, but allowed to recover at 37°C for 10 minutes before further processing. ^86^Rb^+^ was employed as a congener for K^+^. For efflux experiments, the choroidal tissue accumulated radioactive isotopes for 10 minutes (^86^Rb^+^ (1 µCi mL^−1^, 022‐105721‐00321‐0001, POLATOM) and ^3^H‐mannitol (4 µCi mL^−1^, NET101, Perkin Elmer)) with inclusion of bumetanide (20 µm) or vehicle (DMSO)). The choroid plexus was then washed for 5 seconds in isotope‐free HEPES‐aCSF and sequentially transferred to new wells containing 200 µL HEPES‐aCSF with bumetanide or vehicle for 20 seconds. To test the effect of extracellular [K^+^] on the ^86^Rb^+^ efflux rate, the [K^+^] in these wells were 1.5, 2.5, 5, or 10 mm (equimolar replacement with [Na^+^] to retain the osmolarity). The content of each well was collected in scintillation vials and the choroid plexus was placed in a scintillation vial containing 200 µL Solvable (6NE9100, PerkinElmer). Two mL Ultima Gold XR scintillation liquid (6 013 119, Perkin Elmer) was added to all vials and the isotope content measured with a Tri‐Carb 2900TR Liquid Scintillation Analyzer (Packard). The content of ^86^Rb^+^was corrected for extracellular background using ^3^H‐mannitol and the natural logarithm of the ^86^Rb^+^content at each time point (A_t_) normalized to the initial content in the choroid plexus (A_0_) was plotted as a function of time. The ^86^Rb^+^efflux rate constant (min^−1^) was determined with linear regression on the slopes.^[^ ^35^, ^36^ ^]^ For influx experiments, the choroid plexus was treated with either bumetanide (20 µM) or equivalent amount of vehicle (DMSO), or ouabain (2 mm, Sigma, O3125) or vehicle (aCSF) in HEPES‐aCSF for 10 minutes followed by 2 minutes of isotope accumulation in a similar solution with ^86^Rb^+^ (1 µCi mL^−1^) and ^3^H‐mannitol (4 µCi mL^−1^) and a [K^+^] of 1.5, 2.5, 5, or 10 mm (equimolar replacement with [Na^+^] to retain the osmolarity). The 2 min time point was previously demonstrated to be within the time‐linear part of the uptake curve.^[^ ^87^ ^]^ The tissue was then washed twice for 2 sec in ice‐cold HEPES‐aCSF containing 2 mm ouabain, 20 µm bumetanide and 1 mm BaCl_2_ (to minimize loss of accumulated ^86^Rb^+^) and dissolved in 100 µL Solvable. When the tissue was completely dissolved, 80 µL of the solution was transferred to a scintillation vial and isotope content determined as above. When choroid plexus from either the control or the inhibitor group was lost during the experiment (Figures 4B and 5 mm), the value was excluded from the bumetanide‐sensitive efflux.

### Patients

Patients with subarachnoid hemorrhage who were treated with extraventricular drain (EVD) between June 2019 and September 2021 at the Department of Neurosurgery, The Neuroscience Centre, Copenhagen University Hospital, Rigshospitalet, Copenhagen, Denmark, were retrospectively included in the present study, which used CSF samples stored as part of a prospective trial on EVD weaning (clinical trial identifier: NCT03948256^[^ ^88^ ^]^). Only adult patients with CSF samples obtained at the time of placement of the EVD (see below) were included in the study, and all patients had acute hydrocephalus, as clinically evaluated with computed tomography scanning. CSF samples were collected from a total of 32 patients with subarachnoid hemorrhage (mean age: 60 y; range: 27–77 y; F/M: 20/12) through their EVD into a sterile collection tube. The first CSF sample (“start sample”, ≈5 mL CSF) was obtained in the acute phase after placement of the EVD, either within 24 h of ictus (*n* = 21) or as soon as possible hereafter as a sample taken directly from the EVD system (*n* = 11, mean: 44 h; range: 25–74 h). The last CSF sample (“end sample”, 1–2 mL CSF) was obtained either just before the removal of the EVD or during the placement of a ventriculo‐peritoneal shunt. The interval between the start and end samples was on average 19 days, with a range of 5–45 days. After collection, the CSF samples were immediately centrifuged at 2000 × *g* for 10 min, and the supernatant aliquoted in polypropylene microtubes (Sarstedt, Nümbrecht, Germany) before storage at −80 °C.^[^ ^89^ ^]^ Of the 32 patients with  subarachnoid hemorrhage, 20 patients were successfully weaned off the EVD (“weaned”), while the remaining 12 patients underwent ventriculoperitoneal shunt surgery due to chronic PHH (“shunt”). The control group consisted of 14 patients with unruptured aneurysms undergoing preventive surgery (vascular clipping) (mean age: 61 y, range: 39–71 y, 8F/6 M), from whom CSF was collected from the basal cisterns during surgery prior to clipping of the aneurysm. [K^+^]_CSF_ was quantified in a blinded manner with an ABL90 FLEX blood gas analyzer (Radiometer, Copenhagen, Denmark). The study was approved by the Ethics committee of the Capital Region of Denmark (H‐19001474, 14 March 2019 and H‐17011472/69 197, 22 March 2019) and the Danish Data Protection Agency (VD‐2019‐210, 8 April 2019). Oral and written informed consent were obtained from all patients or their next of kin depending on the capacity of the patients and all relevant ethical regulations were complied with. No selection was made based on sex or any social categorization and data were not analyzed according to sex of the subjects. Aliquots of the CSF samples were analyzed for other components in unrelated studies.^[^ ^75^, ^81^, ^90^, ^91^ ^]^

### Statistics

Data analysis and statistical tests were performed with GraphPad Prism 10 (GraphPad Software, La Jolla, US). Data were tested with parametric or non‐parametric tests as appropriate (based on distribution and homogeneity of variance). The statistical analyses were done with Student's t‐test or one‐way ANOVA with either Sidak's or Tukey's multiple comparisons test, as indicated in figure legends. Data are presented as mean ± SEM with significance set at *P* < 0.05 and indicated on figures as ^*^ *P* < 0.05, ^**^ *P* < 0.01, ^***^ *P* < 0.001.

## Conflict of Interest

The authors declare no conflict of interest.

## Author Contributions

N.M., D.B., D.B.J., A.T.S., J.S., H.D., and C.A.H. designed the research; D.B., D.B.J., T.L.TB., J.S., S.N. conducted experiments, M.J., M.H.O., T.C., N.H.N. collected patients samples, N.M., T.L.T‐B. drafted the manuscript. All authors participated in the finalization of the manuscript and approved the final version.

## Supporting information



Supporting Information

## Data Availability

The data that support the findings of this study are available from the corresponding author upon reasonable request.

## References

[1] R. C. Rubin , E. S. Henderson , A. K. Ommaya , M. D. Walker , D. P. Rall , J. Neurosurg. 1966, 25, 430. 5925714 10.3171/jns.1966.25.4.0430

[2] W. E. Dandy , Ann. Surg. 1919, 70, 129. 17864139 10.1097/00000658-191908000-00001 PMC1410318

[3] J. de Rougemont , A. Ames III , F. B. Nesbett , H. F. Hofmann , J. Neurophysiol. 1960, 23, 485. 13743963 10.1152/jn.1960.23.5.485

[4] N. MacAulay , R. F. Keep , T. Zeuthen , Fluids Barriers CNS 2022, 19, 26. 35317823 10.1186/s12987-022-00323-1 PMC8941821

[5] K. Welch , Am. J. Physiol. 1963, 205, 617. 14065919 10.1152/ajplegacy.1963.205.3.617

[6] D. Barbuskaite , E. K. Oernbo , J. H. Wardman , T. L. Toft‐Bertelsen , E. Conti , S. N. Andreassen , N. J. Gerkau , C. R. Rose , N. MacAulay , Fluids Barriers CNS 2022, 19, 53. 35768824 10.1186/s12987-022-00348-6 PMC9245291

[7] E. Carrion , J. H. Hertzog , M. D. Medlock , G. J. Hauser , H. J. Dalton , Arch. Dis. Child. 2001, 84, 68. 11124792 10.1136/adc.84.1.68 PMC1718615

[8] G. Gucer , L. Viernstein , J Neurosurg 1978, 49, 256. 671078 10.3171/jns.1978.49.2.0256

[9] E. K. Oernbo , A. B. Steffensen , P. Razzaghi Khamesi , T. L. Toft‐Bertelsen , D. Barbuskaite , F. Vilhardt , N. J. Gerkau , K. Tritsaris , A. H. Simonsen , S. D. Lolansen , S. N. Andreassen , S. G. Hasselbalch , T. Zeuthen , C. R. Rose , V. Kurtcuoglu , N. MacAulay , Fluids Barriers CNS 2022, 19, 65. 36038945 10.1186/s12987-022-00358-4 PMC9422132

[10] J. Andersson , M. Rosell , K. Kockum , O. Lilja‐Lund , L. Soderstrom , K. Laurell , PLoS One 2019, 14, e0217705. 31141553 10.1371/journal.pone.0217705 PMC6541279

[11] R. Martin‐Laez , H. Caballero‐Arzapalo , L. A. Lopez‐Menendez , J. C. Arango‐Lasprilla , A. Vazquez‐Barquero , World Neurosurg. 2015, 84, 2002. 26183137 10.1016/j.wneu.2015.07.005

[12] E. K. Persson , S. Anderson , L. M. Wiklund , P. Uvebrant , Childs Nerv. Syst. 2007, 23, 1111. 17429657 10.1007/s00381-007-0324-7

[13] M. C. Dewan , A. Rattani , R. Mekary , L. J. Glancz , I. Yunusa , R. E. Baticulon , G. Fieggen , J. C. Wellons , K. B. Park , B. C. Warf , J. Neurosurg. 2018, 130, 1065. 29701543 10.3171/2017.10.JNS17439

[14] S. P. Mollan , M. Aguiar , F. Evison , E. Frew , A. J. Sinclair , Eye 2019, 33, 478. 30356129 10.1038/s41433-018-0238-5 PMC6460708

[15] K. M. Laurence , S. Coates , Arch. Dis. Child. 1962, 37, 345. 14462827 10.1136/adc.37.194.345 PMC2012878

[16] M. Ziebell , J. Wetterslev , M. Tisell , C. Gluud , M. Juhler , Cochrane Database Syst. Rev. 2013, 5, CD009706. 10.1002/14651858.CD009706.pub2 PMC11623054 23728696

[17] I. K. Pople , J. Neurol. Neurosurg. Psychiatry 2002, 73, i17. 12185257 10.1136/jnnp.73.suppl_1.i17 PMC1765598

[18] D. Ben‐Israel , J. A. Mann , M. M. H. Yang , A. M. Isaacs , M. Cadieux , N. Sader , S. Muram , A. Albakr , B. Manoranjan , R. W. Yu , B. Beland , M. G. Hamilton , E. Spackman , P. E. Ronksley , J. Riva‐Cambrin , J. Neurosurg. Pediatr. 2022, 30, 18. 35523256 10.3171/2022.3.PEDS21512

[19] N. Mansoor , O. Solheim , O. A. Fredriksli , S. Gulati , Brain Behav. 2021, 11, e2390. 34661978 10.1002/brb3.2390 PMC8613436

[20] G. K. Reddy , P. Bollam , G. Caldito , World Neurosurg. 2014, 81, 404. 23380280 10.1016/j.wneu.2013.01.096

[21] N. E. Jakopin , E. Myong , T. Bogucki , D. Gray , P. Gross , J. G. McComb , C. N. Shannon , M. S. Tamber , M. Toyama , T. van der Willigen , A. Yazdani , M. G. Hamilton , J. E. Koschnitzky , J. Neurosurg. 2022, 139, 492. 36681979 10.3171/2022.10.JNS22753

[22] T. Speake , L. J. Freeman , P. D. Brown , Biochim. Biophys. Acta 2003, 1609, 80. 12507761 10.1016/s0005-2736(02)00658-2

[23] K. Oshio , H. Watanabe , Y. Song , A. S. Verkman , G. T. Manley , FASEB J. 2005, 19, 76. 15533949 10.1096/fj.04-1711fje

[24] T. Ma , B. Yang , A. Gillespie , E. J. Carlson , C. J. Epstein , A. S. Verkman , J. Biol. Chem. 1998, 273, 4296. 9468475 10.1074/jbc.273.8.4296

[25] S. Chretien , J. P. Catron , Blood 1999, 93, 4021. 10383192

[26] G. M. Preston , B. L. Smith , M. L. Zeidel , J. J. Moulds , P. Agre , Science 1994, 265, 1585. 7521540 10.1126/science.7521540

[27] S. R. Heisey , D. Held , J. R. Pappenheimer , Am. J. Physiol. 1962, 203, 775. 13953498 10.1152/ajplegacy.1962.203.5.775

[28] G. M. Hochwald , A. Wald , J. DiMattio , C. Malhan , Life Sci. 1974, 15, 1309. 4549978 10.1016/0024-3205(74)90312-9

[29] A. Sahar , E. Tsipstein , Exp. Neurol. 1978, 60, 584. 680059 10.1016/0014-4886(78)90012-2

[30] H. H. Damkier , P. D. Brown , J. Praetorius , Physiol Rev. 2013, 93, 1847. 24137023 10.1152/physrev.00004.2013

[31] S. Jacobs , E. Ruusuvuori , S. T. Sipila , A. Haapanen , H. H. Damkier , I. Kurth , M. Hentschke , M. Schweizer , Y. Rudhard , L. M. Laatikainen , J. Tyynela , J. Praetorius , J. Voipio , C. A. Hubner , Proc. Natl Acad. Sci. USA 2008, 105, 311. 18165320 10.1073/pnas.0705487105 PMC2224208

[32] S. Javaheri , K. R. Wagner , J. Clin. Invest. 1993, 92, 2257. 8227341 10.1172/JCI116829 PMC288406

[33] J. K. Karimy , J. Zhang , D. B. Kurland , B. C. Theriault , D. Duran , J. A. Stokum , C. G. Furey , X. Zhou , M. S. Mansuri , J. Montejo , A. Vera , M. L. DiLuna , E. Delpire , S. L. Alper , M. Gunel , V. Gerzanich , R. Medzhitov , J. M. Simard , K. T. Kahle , Nat. Med. 2017, 23, 997. 28692063 10.1038/nm.4361

[34] N. MacAulay , Nat. Rev. Neurosci. 2021, 22, 326. 33846637 10.1038/s41583-021-00454-8

[35] A. B. Steffensen , E. K. Oernbo , A. Stoica , N. J. Gerkau , D. Barbuskaite , K. Tritsaris , C. R. Rose , N. MacAulay , Nat. Commun. 2018, 9, 2167. 29867199 10.1038/s41467-018-04677-9 PMC5986890

[36] R. F. Keep , J. Xiang , A. L. Betz , Am. J. Physiol. 1994, 267, C1616. 7810603 10.1152/ajpcell.1994.267.6.C1616

[37] N. MacAulay , C. R. Rose , J. Physiol., 2020, 598, 4737. 32870507 10.1113/JP279868

[38] N. MacAulay , C. R. Rose , J. Physiol., 2020, 598; 4743. 32870504 10.1113/JP280494

[39] F. J. Alvarez‐Leefmans , J. Physiol., 2020, 598; 4733. 32870510 10.1113/JP279867

[40] F. J. Alvarez‐Leefmans, J. Physiol., 2020, 598; 4741. 32870502 10.1113/JP280493

[41] H. Xu , R. M. Fame , C. Sadegh , J. Sutin , C. Naranjo , S. Della , J. Cui , F. B. Shipley , A. Vernon , F. Gao , Y. Zhang , M. J. Holtzman , M. Heiman , B. C. Warf , P. Y. Lin , M. K. Lehtinen , Nat. Commun. 2021, 12, 447. 33469018 10.1038/s41467-020-20666-3 PMC7815709

[42] Y. Hua , X. Ying , Y. Qian , H. Liu , Y. Lan , A. Xie , X. Zhu , Biosci. Rep. 2019, 39, BSR20182303. 31023968 10.1042/BSR20182303 PMC6522737

[43] C. L. Chou , M. A. Knepper , A. N. Hoek , D. Brown , B. Yang , T. Ma , A. S. Verkman , J. Clin. Invest. 1999, 103, 491. 10021457 10.1172/JCI5704 PMC408109

[44] J. Schnermann , C. L. Chou , T. Ma , T. Traynor , M. A. Knepper , A. S. Verkman , Proc. Natl Acad. Sci. U.S.A. 1998, 95, 9660. 9689137 10.1073/pnas.95.16.9660 PMC21395

[45] X. Chen , Y. He , Y. Tian , Y. Wang , Z. Wu , T. Lan , H. Wang , K. Cheng , P. Xie , Hum. Gene Ther. 2020, 31, 440. 32056463 10.1089/hum.2019.300

[46] L. Zhu , L. R. Stein , D. Kim , K. Ho , G. Q. Yu , L. Zhan , T. E. Larsson , L. Mucke , Proc. Natl. Acad. Sci. U.S.A. 2018, 115, E11388. 30413620 10.1073/pnas.1808609115 PMC6275534

[47] C. H. Mazucanti , V. Kennedy Jr. , H. U. Premathilake , M. E. Doyle , J. Tian , Q. R. Liu , J. O'Connell , S. Camandola , J. M. Egan , Cell Rep. 2023, 42, 112903. 37515772 10.1016/j.celrep.2023.112903 PMC10529429

[48] E. Delpire , K. B. Gagnon , Am. J. Physiol. Cell Physiol. 2019, 316, C522. 30576234 10.1152/ajpcell.00490.2018 PMC6482669

[49] S. N. Andreassen , T. L. Toft‐Bertelsen , J. H. Wardman , R. Villadsen , N. MacAulay , Fluids Barriers CNS 2022, 19, 44. 35659263 10.1186/s12987-022-00335-x PMC9166438

[50] J. Farinas , M. Kneen , M. Moore , A. S. Verkman , J. Gen. Physiol. 1997, 110, 283. 9276754 10.1085/jgp.110.3.283 PMC2229369

[51] K. Welch , K. Sadler , G. Gold , Am. J. Physiol. 1966, 210, 232. 5901459 10.1152/ajplegacy.1966.210.2.232

[52] S. D. Lolansen , N. Rostgaard , T. Capion , N. H. Norager , M. H. Olsen , M. Juhler , T. I. Mathiesen , N. MacAulay , Int. J. Mol. Sci. 2023, 24,. 10.3390/ijms241411476 PMC10380704 37511234

[53] E. K. Oernbo , A. B. Steffensen , H. Gredal , H. H. Poulsen , N. Rostgaard , C. H. Rasmussen , M. Moller‐Nissen , A. H. Simonsen , S. G. Hasselbalch , M. Juhler , N. MacAulay , Fluids Barriers CNS 2022, 19, 52. 35761330 10.1186/s12987-022-00349-5 PMC9238121

[54] P. Razzaghi Khamesi , V. Charitatos , E. K. Heerfordt , N. MacAulay , V. Kurtcuoglu , Fluids Barriers CNS 2023, 20, 18. 36915140 10.1186/s12987-023-00419-2 PMC10012606

[55] R. Green , G. Giebisch , R. Unwin , A. M. Weinstein , Am. J. Physiol. 1991, 261, F1046. 1750518 10.1152/ajprenal.1991.261.6.F1046

[56] D. S. Parsons , D. L. Wingate , Biochim. Biophys. Acta 1961, 46, 170. 13733011 10.1016/0006-3002(61)90660-6

[57] E. W. Reid , Br. Med. J. 1892, 1, 1133. 10.1136/bmj.1.1639.1133 PMC2420390 20753720

[58] J. Praetorius , S. Nielsen , Am. J. Physiol. Cell Physiol. 2006, 291, C59. 16481371 10.1152/ajpcell.00433.2005

[59] S. Hamann , J. J. Herrera‐Perez , T. Zeuthen , F. J. Alvarez‐Leefmans , J. Physiol. 2010, 588, 4089. 20819947 10.1113/jphysiol.2010.194738 PMC3002443

[60] T. Zeuthen , N. MacAulay , J.Physiol 2012, 590, 1139. 22250214 10.1113/jphysiol.2011.226316 PMC3381821

[61] T. Zeuthen , J. Membr. Biol. 2010, 234, 57. 20091162 10.1007/s00232-009-9216-y

[62] T. Zeuthen , A. B. Steffensen in Role of the Choroid Plexus in Health and Disease (Eds. B. Blazer‐Yost , J. Praetorius , H. Damkier ), Springer, Berlin 2020.

[63] M. W. Bradbury , C. R. Kleeman , Am. J. Physiol. 1967, 213, 519. 6036340 10.1152/ajplegacy.1967.213.2.519

[64] R. Katzman , Fed. Proc. 1976, 35, 1244. 770198

[65] M. W. Bradbury , B. Stulcova , J. Physiol. 1970, 208, 415. 5500733 10.1113/jphysiol.1970.sp009128 PMC1348757

[66] G. W. Goldstein , J. Physiol. 1979, 286, 185. 439024 10.1113/jphysiol.1979.sp012613 PMC1281565

[67] K. Lykke , M. Assentoft , S. Horlyck , H. C. Helms , A. Stoica , T. L. Toft‐Bertelsen , K. Tritsaris , F. Vilhardt , B. Brodin , N. MacAulay , J. Cereb. Blood. Flow Metab. 2019, 497. 28994331 10.1177/0271678X17736715 PMC6421245

[68] J. T. Parmelee , D. Bairamian , C. E. Johanson , Brain Res. Dev. Brain Res. 1991, 60, 229. 1654233 10.1016/0165-3806(91)90051-j

[69] N. MacAulay , T. L. Toft‐Bertelsen , Cell Calcium 2023, 116, 102797. 37801806 10.1016/j.ceca.2023.102797

[70] K. T. Lu , C. Y. Wu , N. C. Cheng , Y. Y. Wo , J. T. Yang , H. H. Yen , Y. L. Yang , Eur. J. Pharmacol. 2006, 548, 99. 16962576 10.1016/j.ejphar.2006.07.048

[71] T. Metayer , C. Orset , C. Ali , J. Furon , N. Szabla , E. Emery , D. Vivien , T. Gaberel , Acta Neurochir. 2022, 164, 499. 35094147 10.1007/s00701-021-05088-4

[72] X. J. Chen , N. Kovacevic , N. J. Lobaugh , J. G. Sled , R. M. Henkelman , J. T. Henderson , Neuroimage 2006, 29, 99. 16084741 10.1016/j.neuroimage.2005.07.008

[73] O. Natt , T. Watanabe , S. Boretius , J. Radulovic , J. Frahm , T. Michaelis , J. Neurosci. Methods 2002, 120, 203. 12385770 10.1016/s0165-0270(02)00211-x

[74] S. D. Lolansen , D. Barbuskaite , F. Ye , J. Xiang , R. F. Keep , N. MacAulay , Fluids Barriers CNS 2023, 20, 53. 37403103 10.1186/s12987-023-00448-x PMC10318838

[75] T. L. Toft‐Bertelsen , D. Barbuskaite , E. K. Heerfordt , S. D. Lolansen , S. N. Andreassen , N. Rostgaard , M. H. Olsen , N. H. Norager , T. Capion , M. F. Rath , M. Juhler , N. MacAulay , Fluids Barriers CNS 2022, 19, 69. 36068581 10.1186/s12987-022-00361-9 PMC9450297

[76] J. H. Wardman , S. N. Andreassen , T. L. Toft‐Bertelsen , M. N. Jensen , J. E. Wilhjelm , B. Styrishave , S. Hamann , S. Heegaard , A. J. Sinclair , N. MacAulay , Fluids Barriers CNS 2024, 21, 10. 38273331 10.1186/s12987-024-00511-1 PMC10810013

[77] J. H. Wardman , M. N. Jensen , S. N. Andreassen , B. Styrishave , J. E. Wilhjelm , A. J. Sinclair , N. MacAulay , Fluids Barriers CNS 2023, 20, 44. 37328884 10.1186/s12987-023-00436-1 PMC10276479

[78] S. M. Robert , B. C. Reeves , E. Kiziltug , P. Q. Duy , J. K. Karimy , M. S. Mansuri , A. Marlier , G. Allington , A. B. W. Greenberg , T. DeSpenza Jr. , A. K. Singh , X. Zeng , K. Y. Mekbib , A. J. Kundishora , C. Nelson‐Williams , L. T. Hao , J. Zhang , T. T. Lam , R. Wilson , W. E. Butler , M. L. Diluna , P. Feinberg , D. P. Schafer , K. Movahedi , A. Tannenbaum , S. Koundal , X. Chen , H. Benveniste , D. D. Limbrick Jr. , S. J. Schiff , et al., Cell 2023, 186, 764. 36803604 10.1016/j.cell.2023.01.017 PMC10069664

[79] W. Zhang , M. Freichel , F. van der Hoeven , P. P. Nawroth , H. Katus , F. Kalble , E. Zitron , V. Schwenger , PLoS One 2016, 11, e0145513. 26760974 10.1371/journal.pone.0145513 PMC4711985

[80] M. W. Antoine , C. A. Hubner , J. C. Arezzo , J. M. Hebert , Science 2013, 341, 1120. 24009395 10.1126/science.1240405 PMC4731229

[81] S. D. Lolansen , N. Rostgaard , D. Barbuskaite , T. Capion , M. H. Olsen , N. H. Norager , F. Vilhardt , S. N. Andreassen , T. L. Toft‐Bertelsen , F. Ye , M. Juhler , R. F. Keep , N. MacAulay , Fluids Barriers CNS 2022, 19, 62. 35948938 10.1186/s12987-022-00360-w PMC9367104

[82] H. Tanioka , T. Shirakawa , T. Machida , Y. Sasaki , Radiology 1991, 178, 141. 1984292 10.1148/radiology.178.1.1984292

[83] B. B. Avants , N. J. Tustison , M. Stauffer , G. Song , B. Wu , J. C. Gee , Front. Neuroinform. 2014, 8, 44. 24817849 10.3389/fninf.2014.00044 PMC4009425

[84] N. J. Tustison , B. B. Avants , P. A. Cook , Y. Zheng , A. Egan , P. A. Yushkevich , J. C. Gee , IEEE Trans Med Imaging 2010, 29, 1310. 20378467 10.1109/TMI.2010.2046908 PMC3071855

[85] P. A. Yushkevich , J. Piven , H. C. Hazlett , R. G. Smith , S. Ho , J. C. Gee , G. Gerig , Neuroimage 2006, 31, 1116. 16545965 10.1016/j.neuroimage.2006.01.015

[86] F. Gremse , M. Stark , J. Ehling , J. R. Menzel , T. Lammers , F. Kiessling , Theranostics 2016, 6, 328. 26909109 10.7150/thno.13624 PMC4737721

[87] A. B. Steffensen , B. L. Edelbo , D. Barbuskaite , S. N. Andreassen , M. H. Olsen , K. Moller , N. MacAulay , Fluids Barriers CNS 2023, 20, 49. 37353833 10.1186/s12987-023-00451-2 PMC10290349

[88] Danish Randomized Trial of External Ventricular Drainage Cessation IN Aneurysmal Subarachnoid Haemorrhage (DRAIN), https://clinicaltrials.gov/ct2/show/NCT03948256, (accessed: June 2024).

[89] M. del Campo , B. Mollenhauer , A. Bertolotto , S. Engelborghs , H. Hampel , A. H. Simonsen , E. Kapaki , N. Kruse , N. L. Bastard , S. Lehmann , J. L. Molinuevo , L. Parnetti , A. Perret‐Liaudet , J. Sáez‐Valero , E. Saka , A. Urbani , E. Vanmechelen , M. Verbeek , P. J. Visser , C. Teunissen , Biomark. Med. 2012, 6, 419. 22917144 10.2217/bmm.12.46

[90] N. Rostgaard , M. H. Olsen , T. Capion , N. MacAulay , M. Juhler , Biomedicines 2023, 11, 997. 37189615 10.3390/biomedicines11040997 PMC10135965

[91] T. L. Toft‐Bertelsen , S. N. Andreassen , N. Rostgaard , M. H. Olsen , N. H. Norager , T. Capion , M. Juhler , N. MacAulay , Biomedicines 2023, 11, 2360. 37760800 10.3390/biomedicines11092360 PMC10525923

