# Supplementary material for: The Na+,K+,2Cl− Cotransporter, Not Aquaporin 1, Sustains Cerebrospinal Fluid Secretion While Controlling Brain K+ Homeostasis
Source: Adv Sci (Weinh). 2024 Dec 18;12(6):2409120. doi: 10.1002/advs.202409120 (PMC11809428; doi:10.1002/advs.202409120)
Supplement: Supplementary file 1 — Supporting Information [file ADVS-12-2409120-s001.pdf]

ADVANCED SCIENCE
Open Access

Supporting Information

for *Adv. Sci.*, DOI 10.1002/advs.202409120

The Na^+,K^+,$2Cl^-$ Cotransporter, Not Aquaporin 1, Sustains Cerebrospinal Fluid Secretion While Controlling Brain K^+ Homeostasis

*Dennis Bo Jensen, Trine L. Toft-Bertelsen, Dagne Barbuskaite, Jane Stubbe, Sandor Nietzsche, Tenna Capion, Nicolas H. Norager, Markus H. Olsen, Andreas T. Sørensen, Henrik Dimke, Christian A. Hübner, Marianne Juhler and Nanna MacAulay**

Supplementary figures

The Na^+,K^+,$2Cl^-$ cotransporter, not aquaporin 1, sustains cerebrospinal fluid secretion while controlling brain K^+ homeostasis

*Dennis Bo Jensen, Trine L. Toft-Bertelsen, Dagne Barbuskaite, Jane Stubbe, Sandor Nietzsche, Tenna Capion, Nicolas H. Norager, Markus H. Olsen, Andreas T. Sørensen, Henrik Dimke, Christian A. Hübner, Marianne Juhler, & Nanna MacAulay**

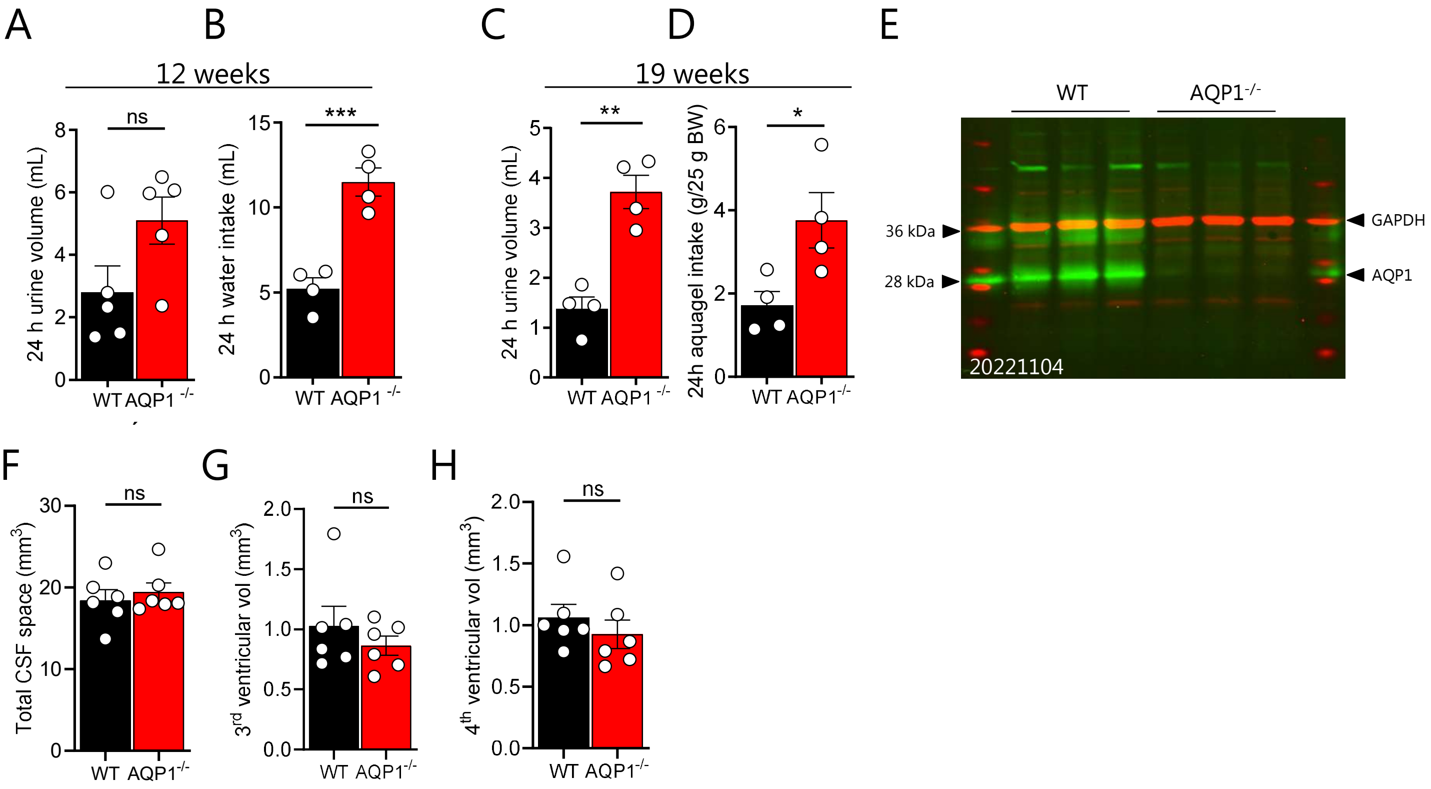

Figure S1 Undisturbed whole body water balance in AQP1$^{-/-}$. A-B 24 h Diuresis and water intake in WT and AQP1$^{-/-}$ 12 weeks old and 19 weeks old (**C-D**) mice (n = 5 for panel A and AQP1$^{-/-}$ in panel B and n = 4 in the remaining panels). One statistically defined outlier was removed from the WT group in panel B. **E** Western blotting of choroid plexus lysates from WT and AQP1$^{-/-}$ mice with AQP1 in green (25 kDa) and GAPDH in red (37 kDa), n = 3. **F-H** Quantification of CSF spaces from MRI with total CSF space (**F**), 3rd (**G**) and 4th (**H**) ventricular volume in WT and AQP1$^{-/-}$ mice, n = 6 of each. Statistical evaluation with Student't t-test. *$P < 0.05$; **$P < 0.01$; ***$P < 0.001$; ns: not significant.

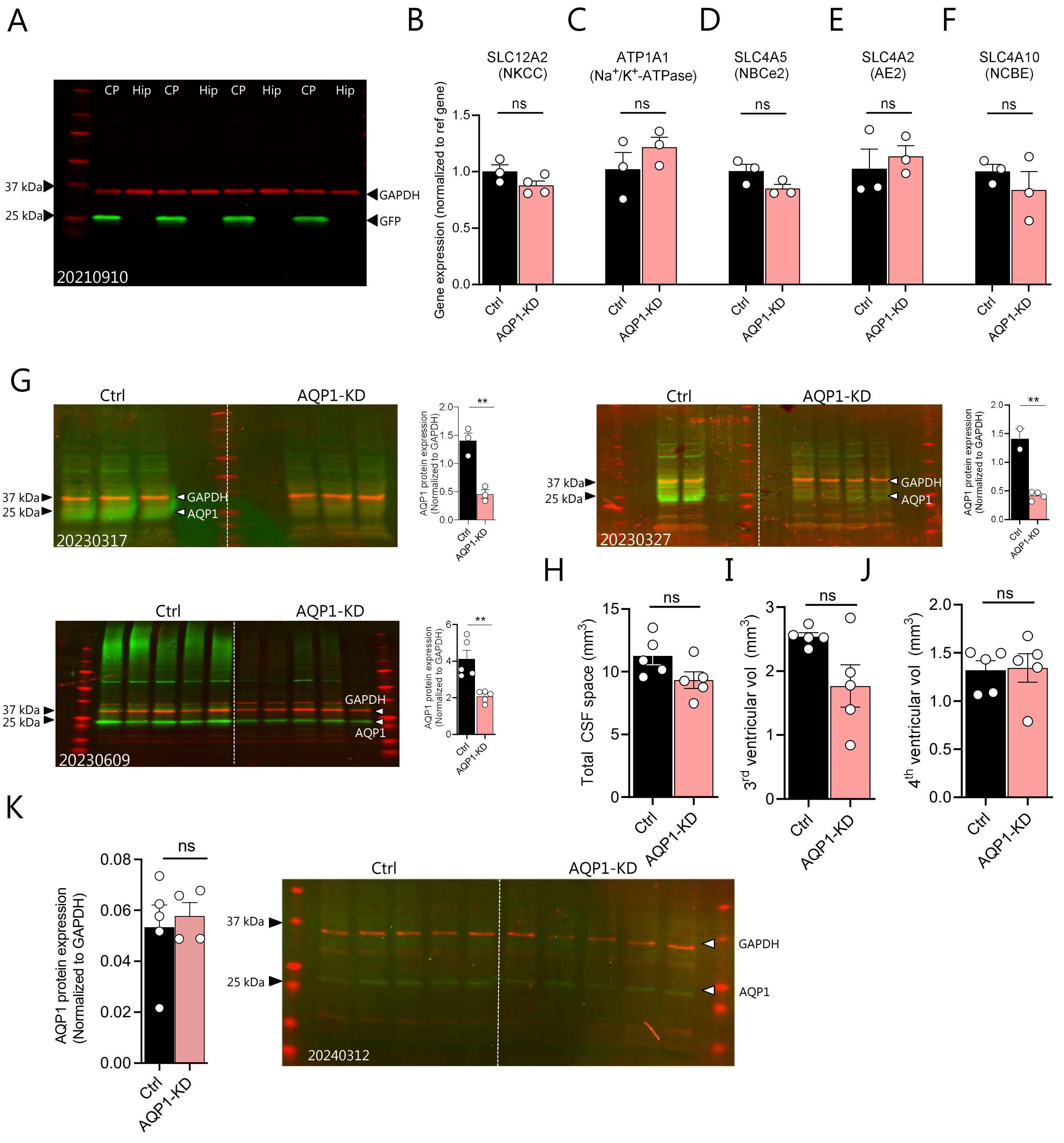

Figure S2 Viral knock-down of AQP1. A Western blotting of choroid plexus (CP) and hippocampal (hip) lysates from AAV-GFP-injected mice, n = 4. **B** qPCR-based gene expression of SLC12A2, **C** ATP1A1, **D** SLC4A5, **E** SLC4A2 and **F** SLC4A10 in mouse choroid plexus from AAV-GFP-injected (Ctrl) and AAV-Cre-injected (AQP1-KD) $AQP1^{flox/flox}$ mice, n = 4 $AQP1^{-/-}$ in panel B and n = 3 in remaining panels. **G** Western blotting of choroid plexus from AAV-GFP-injected mice (Ctrl) vs AAV-Cre-injected mice (AQP1-KD) three weeks post-injection (n = 3 independent experiments, each with 2-5 biological replicates). **H-J** Quantification of CSF spaces from MRI with total CSF space (**H**), 3rd (**I**) and 4th (**J**) ventricular volume in AAV-GFP-injected- mice (Ctrl) and AAV-Cre-injected mice (AQP1-KD), n = 5 of each. **K** Western blotting of kidney tissue from AAV-GFP-injected mice (Ctrl) and AAV-Cre-injected mice (AQP1-KD), n = 5 control and n = 4 AQP1-KD, the latter with one statistical outlier (Grubb's test). Statistical evaluation with one-way ANOVA with Sidak's post-hoc test (panel B) or Student't t-test (remaining panels). *P < 0.0; **P < 0.01; ns: not significant.

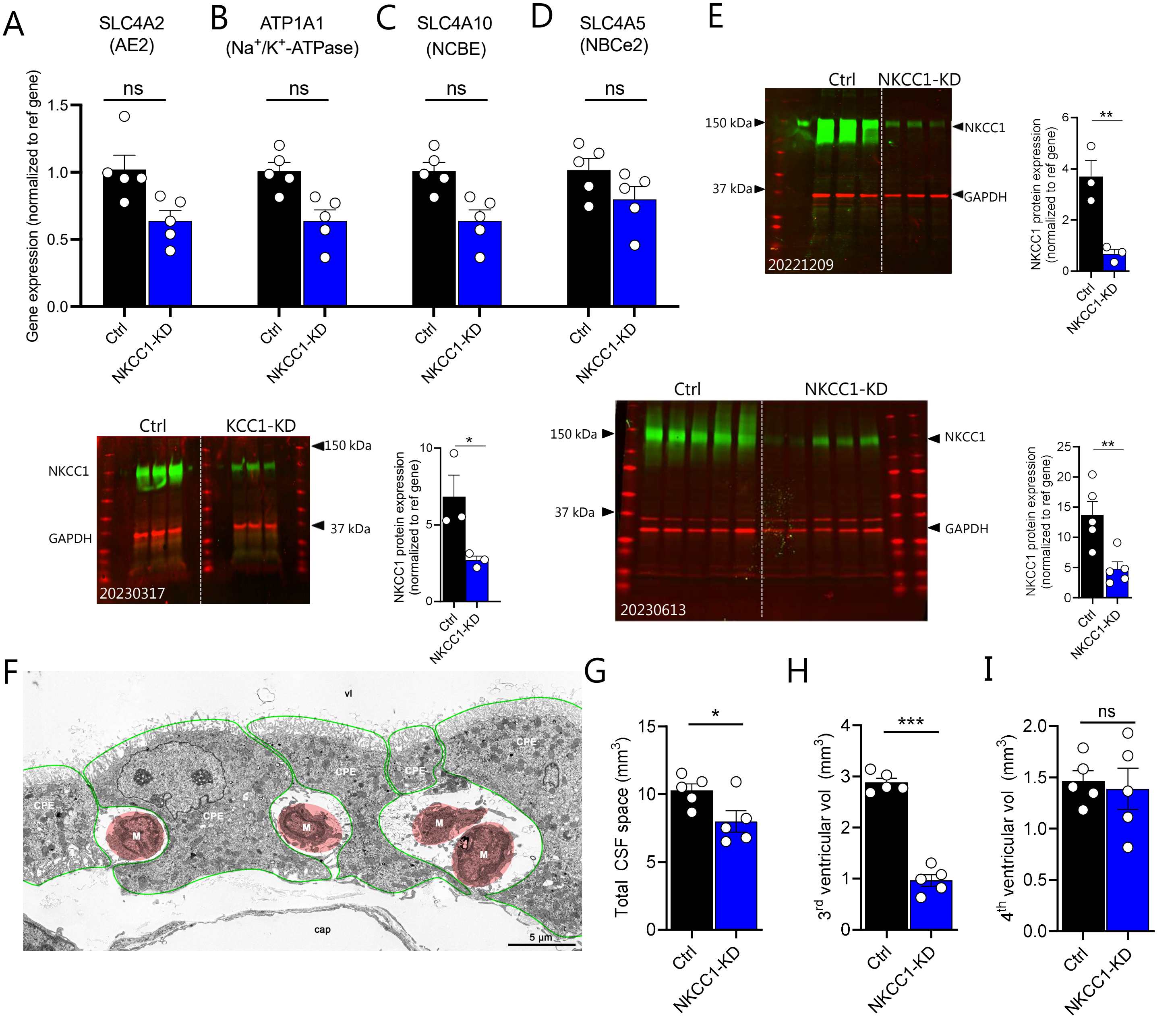

Figure S3 Viral knock-down of NKCC1. **A** qPCR-based Gene expression of SLC4A2, **B** ATP1A1, **C** SLC4A10 and **D** SLC4A5 in mouse choroid plexus from AAV-GFP-injected (Ctrl) and AAV-Cre-injected (NKCC1-KD) NKCC1$^{flox/flox}$ mice, n = 5 of each. **E** Western blotting of choroid plexus from AAV-GFP-injected (Ctrl) mice vs AAV-Cre-injected mice (NKCC1-KD) three weeks post-injection (n = 3 independent experiments, each with 3-5 biological replicates). **F** Electron micrograph illustrating infiltrating migrating cells in the stroma of the NKCC1-KD mice (three weeks post-injection with AAV-cre). Choroid plexus epithelial cells are lined in green and migrating cells highlighted in red; vl: ventricular lumen; cap: blood capillary). **G-I** Quantification of CSF spaces from MRI with total CSF space (**G**), 3rd ventricular volume (**H**) and 4th ventricular volume (**I**) in AAV-GFP-injected (Ctrl) and AAV-Cre-injected (NKCC1-KD) SLC12A2$^{flox/flox}$ mice, n = 5 of each. Statistical evaluation with with one-way ANOVA with Sidak's post-hoc test (panel A) or Student't t-test (remaining panels). *$P < 0.05$; **$P<0.001$; ***$P < 0.001$; ns: not significant.

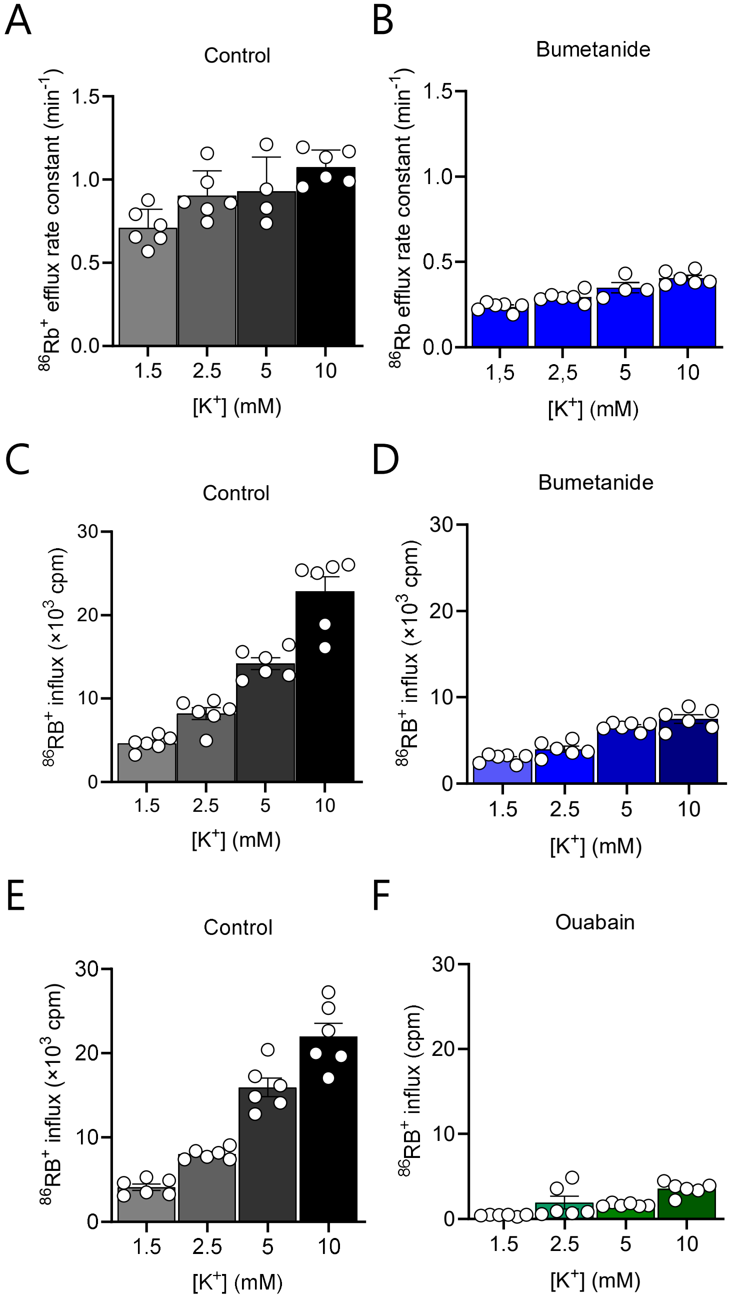

Figure S4 ^{86}Rb^{+} flux assays in control solution and with inclusion of inhibitors. A ^{86}Rb^{+} efflux rate from pre-equilibrated choroid plexus in control solution and (**B**) upon inclusion of the NKCC1 inhibitor bumetanide (20 µM) with increasing [K^{+}] in the test solution (1.5 - 10 mM), n = 6 of each, except 5 mM, where one choroid plexus in each group was lost during experimentation. **C** ^{86}Rb^{+} influx (two min) in choroid plexus in control solution and (**D**) upon inclusion of the NKCC1 inhibitor bumetanide (20 µM) with increasing [K^{+}] in the test solution (1.5 - 10 mM), n = 6 of each. **E** ^{86}Rb^{+} influx (two min) in choroid plexus in control solution and (**F**) upon inclusion of the Na^{+}/K^{+}-ATPase inhibitor ouabain (2 mM) with increasing [K^{+}] in the test solution (1.5 - 10 mM), n = 6 of each. All values represent one lateral choroid plexus.
